# Novel vaccination strategies based on optimal stimulation of CD4^+^ T helper cells for the treatment of oral squamous cell carcinoma

**DOI:** 10.3389/fimmu.2024.1387835

**Published:** 2024-07-05

**Authors:** Lorenzo Azzi, Fabrizio Celesti, Anna Maria Chiaravalli, Amruth Kaleem Basha Shaik, Mariam Shallak, Andrea Gatta, Paolo Battaglia, Stefano La Rosa, Angelo Tagliabue, Roberto Sergio Accolla, Greta Forlani

**Affiliations:** ^1^ Department of Medicine and Technological Innovation, University of Insubria, Varese, Italy; ^2^ Azienda Socio-Sanitaria Territoriale (ASST) dei Sette Laghi, Varese, Italy; ^3^ Center for Immuno-Oncology, Department of Medicine, Surgery, and Neurosciences, University of Siena, Siena, Italy; ^4^ Department of Biotechnology and Life Sciences, University of Insubria, Varese, Italy; ^5^ Azienda Socio-Sanitaria Territoriale (ASST) Lariana, San Fermo della Battaglia, CO, Italy; ^6^ Department of Medicine and Surgery, University of Insubria, Varese, Italy

**Keywords:** CIITA, tumor vaccination, T helper, MHC-II, oral squamous cell carcinoma, oral cancer, adaptive immunity

## Abstract

Oral Squamous Cell Carcinoma (OSCC) is the most common malignant tumor of the oral cavity. Despite recent advances in the field of oral cancer therapy, including the introduction of immunotherapeutic approaches, the 5-year survival rate remains steadily assessed around 50%. Thus, there is an urgent need for new therapeutic strategies. After the characterization of the immune phenotype of three human OSCC cell lines (CAL-27, SCC-25, and SCC-4) and one mouse OSCC cell line (MOC2) showing their similarities to resected patient tumors, we explored for the first time an experimental preclinical model of therapeutic vaccination with mouse OSCC MOC2 cell line stably expressing MHC class II antigens after CIITA gene transfection (MOC2-CIITA). Mice injected with MOC2-CIITA reject or strongly retard tumor growth; more importantly, vaccinated animals that fully reject MOC2-CIITA tumors display anti-tumor immunological memory protective against challenge with parental MOC2 tumor cells. Further experiments of adoptive cell transfer or *in vivo* cell depletion show that both CD4^+^ and CD8^+^ T lymphocytes prove fundamental in tumor rejection. This unprecedented approach for oral cancer opens the way for possible future translation of novel immunotherapeutic strategies to the human setting for the treatment of this tumor.

## 1 Introduction

Oral Squamous Cell Carcinoma (OSCC) accounts for 90% of malignancies of the oral cavity and originates from the malignant transformation of keratinocytes, the cells which constitute the epithelium of the mucosal lining of the mouth (1). With 377,713 new cases recorded in 2020, OSCC represents the 16^th^ most common malignancy worldwide and the most representative in the group of head and neck squamous cell carcinoma (HNSCC) (2). Major risk factors for OSCC are well established in the literature and include tobacco, alcohol, and betel quid consumption, but 10–15% of patients develop cancer even if they have not been exposed to traditional risks (3–6). In addition, there is an alarming increase of the incidence of oral cancer in younger subjects, and this trend is not related to Human Papilloma Virus (HPV) infection as described in Oropharyngeal cancer (7). Current treatment options include surgery, radiotherapy and/or chemotherapy and are associated with significant impairment of the quality of life for survivors. Despite recent advances in the field of cancer therapy, in the last decades the 5-year survival rate of oral cancer remained steadily assessed around 50%, with 177,757 deaths recorded in 2020 (2). Locally advanced and unresectable, recurrent and/or metastatic OSCC shows poor prognosis, with short overall survival (8). New therapeutic strategies are thus urgently needed for this form of cancer.

Recently, immunotherapeutic approaches based on the optimal stimulation of anti-tumor immunity have been considered with particular attention (9). For example, treatments with antibodies specific for immune checkpoints (immune checkpoint inhibitors or ICI) expressed on effector T cells (i.e., programmed cell death protein 1 or PD-1) and antigen presenting cells (APC) and, importantly, often on tumor cells (i.e., programmed death ligand 1 or PD-L1) have obtained unprecedented positive results, particularly in metastatic melanoma and non-small cell lung cancer (NSCLC) (10). However, it is becoming clear that even ICI do not work with all tumors, and within the same tumor they still present unclear variability (11, 12). Additional treatments which would take into account also the initial triggering of the anti-tumor immune response are thus under study (13).

Within this frame, attempts have been made to use peptide vaccines targeted mainly at the stimulation of tumor-specific Major Histocompatibility Complex (MHC) class I-restricted CD8^+^ cytotoxic T lymphocytes (CTL), the major effectors of anti-tumor responses (14). MHC class I (MHC-I) molecules are expressed on the surface of most mammalian cells, including tumors, and play a pivotal role in the presentation of intracellularly synthesized antigens to CTLs (15). Being the CD8^+^ CTLs the final effectors of the adaptive anti-tumor immune response, it is expected that the expression of MHC-I molecules in tumor cells represents one of the most important parameters associated with the efficacy of the immune response itself. Indeed, lack or reduced expression of MHC-I molecules in tumor cells, including the case of OSCC, is often associated with tumor escape from the immune system (16, 17). However, as reported in preliminary studies, MHC-I-based peptide anti-tumor vaccination approach has encountered critical difficulties in stimulating and maintaining the CTL response against tumors (18).

These results suggest reconsidering the main cellular and molecular components that should be primarily activated in an optimal anti-tumor immune response, i.e., the CD4^+^ T helper lymphocytes (TH) (13). Indeed, without triggering of TH cells, a prolonged effector CTLs response cannot be maintained. However, TH cell antigen recognition is restricted by MHC class II (MHC-II) molecules that, differently from MHC-I antigens, are constitutively expressed only on few cell types, particularly B-cells and APC, like dendritic cells and macrophages (19). Of note, other cell types, including tumor cells, may express MHC-II molecules under the induction of inflammatory cytokines, such as interferon-γ (IFN-γ) (20). In tumors, especially in those of epithelial origin, MHC-II expression is not a common event but when present is usually associated with a better clinical prognosis (21). MHC-II expression in cells, including tumor cells, is under the control of the class II transactivator (CIITA), identified for the first time by our research group (22).

Based on this evidence, we previously demonstrated that MHC-II-negative tumors could express MHC-II molecules upon CIITA gene transfection (23, 24). Indeed, it was shown that CIITA-driven MHC-II-expressing tumor cells can play the role of surrogate APC for their own tumor antigens and trigger an adaptive response *in vivo* capable to reject the tumor as result of stimulation of tumor-specific TH cells which in turn is accompanied by a drastic modification of the tumor microenvironment (25).

Toward the goal to apply for OSCC possible strategies of therapeutic vaccination, in this study we preliminary characterized by immunohistochemistry (IHC) the immune component of tumor microenvironment and the immunophenotype of tumor tissues resected in a cohort of OSCC patients who underwent surgical treatment in our Hospital, ASST dei Sette Laghi, Varese. The tumor immunophenotype was compared to the cell surface phenotype of three human OSCC cell lines (CAL-27, SCC-25, SCC-4) and one mouse OSCC cell line (MOC2). We found that the cell lines mirrored the phenotype of OSCC cells observed in patients, particularly as in relation to the expression of MHC-I and MHC-II, as well as PD-1 and PD-L1. These studies were prodromic to investigate for the first time in an animal experimental model of OSCC our approach of tumor vaccination with CIITA-transduced MHC-II-positive tumor cells.

We demonstrate that CIITA-OSCC cells are potent stimulators of an adaptive immune response that protects the mouse from tumor onset or significantly retards tumor growth. Of note, CIITA-OSCC vaccinated animals that reject the tumor develop an anti-tumor immunological memory capable of rejecting or strongly counteracting challenges with parental OSCC tumor cells. Further experiments of adoptive cell transfer or *in vivo* cell depletion showed that both CD4^+^ and CD8^+^ T lymphocytes prove fundamental in tumor rejection.

Taken together, these results represent the first evidence that a protective adaptive immune response against OSCC, the most frequent tumor of the oral cavity, may be elicited *in vivo* by inducing the tumor cells to express MHC-II molecules in a CIITA-dependent manner. Our findings are discussed within the frame of possible novel strategies of combined immunotherapy for OSCC. Indeed, they open the way to possible future translation of this approach to the human setting, alone or in synergy with immune checkpoint blockade for the treatment of oral cancer.

## 2 Materials and methods

### 2.1 Immunohistochemical study

Formalin-fixed paraffin-embedded (FFPE) samples of 23 OSCC patients who underwent surgical treatment from 2008 to 2013 in our university hospital (ASST dei Sette Laghi, Varese) were retrieved from the files of the Unit of Pathology. Included cases were reviewed by two expert pathologists according to the 8^th^ Edition of the American Joint Committee on Cancer (AJCC) Staging Manual and the 5^th^ Edition of the World Health Organization (WHO) Classification of Head and Neck Tumors (26, 27). All included cases were HPV-negative, conventional-type OSCC and provided a sufficient number of tissue slides to perform immunohistochemistry (IHC) reactions in the current study. The protocol was approved by the Institutional Ethical Committee (Comitato Etico dell'Insubria, protocol n° 19/2018).

The following clinical data were retrospectively collected for each included patient: age, sex, cervical lymph node metastasis (LN metastasis) and stage at diagnosis, and 5-year cancer-related death (CRD).

Firstly, we evaluated the degree of tumor lymphoid infiltration in hematoxylin-eosin staining slides by counting the number of lymphocytes infiltrating the tumor epithelial nests per High Power Field (HPF), and a score from 0 to 2 was defined for each case, with score 0 = no or few cells (i.e., less than 5 cells per HPF); 1 = low-moderate number of cells (between 5 and 20 cells per HPF); and 2 = high number of cells (i.e., 20 cells or more per HPF). The immune infiltration was also evaluated in the tumor stroma among epithelial nests and along tumor growth front, and the stromal area occupied by immune cells was scored from 0 to 2, with score 0 = none or low infiltration (less than 1% of stromal area); 1 = moderate infiltration (from 1 to 10%); and 2 = high infiltration (at least 10%).

The presence of T lymphocytes and monocytic-macrophage cells in both tumor epithelial nests and stroma was evaluated with appropriate staining with antibodies for specific markers: anti-CD4^+^ for TH (Ventana, rabbit, clone SP35), anti-CD8^+^ for CTL (Ventana, rabbit, clone SP57), and anti-CD68^+^ for monocyte-macrophages (DAKO, mouse, clone 1G12) (28).

Consistently with our aim, the phenotypic characterization of MHC-I and MHC-II expression was assessed on tumor cell surface and compared with the patients' clinical outcomes and the immune infiltration. In the same manner, the immune checkpoints PD-1 and PD-L1 were measured in tumor and stromal compartments. Of note, PD-L1 expression was measured both by the internationally recognized Combined Positive Score (CPS) and, separately, on immune cells and, when present, on tumor cells by the Tumor Proportion Score (TPS) (29).

IHC stainings were performed on 3 µm FFPE sections deparaffinized and rehydrated through alcohol series to water, as previously described (30). Briefly, endogenous activity was blocked with 3% aqueous hydrogen peroxide for 10 min, antigen retrieval was performed for each antigen in a domestic 750 kW microwave oven with different solutions (EDTA or citrate buffer pH 6.0). Primary antibodies (**Supplementary Table S1**) were applied overnight at 4°C followed by a polymeric detection system (Ultravision DAB Detection System, LabVision, Värmdö, Sweden) according to the manufacturer's instructions. The immunoreaction was developed with 3.3'-diaminobenzidine tetrahydrochloride (DAB) (Sigma Aldrich, St. Louis, MO, USA) as chromogen.

For each case of OSCC, the MHC-I, MHC-II, and PD-L1 expression was evaluated on tumor cells and scored as summarized in **Supplementary Table S2**. Briefly, MHC-I expression was scored as low (< 50% of immunoreactive tumor cells), moderate (immunoreactive cells ≥ 50% and < 90%), and high expression (immunoreactive cells ≥ 90%); MHC-II was scored as expressed when positive tumor cells were at least 10%; PD-L1 Tumor Proportion Score (TPS) was considered positive when immunoreactivity was present in at least 10% of tumor cells; PD-L1 Combined Positive Score (CPS) was considered positive when ≥ 1 and scored as highly expressed when it was at least 10. CD4^+^, CD8^+^, CD68^+^, and PD-1 immunoreactivities in tumor epithelial nests and stroma were categorized into low or high expression as reported in **Supplementary Table S2**.

### 2.2 Cell lines

Three human OSCC cell lines, CAL-27, SCC-25, and SCC-4, were used for the characterization of the cell surface immune phenotype by immunofluorescence and cytofluorimetry. The three cell lines represent epithelial, adherent squamous cell carcinoma cells obtained from patients affected by tongue cancer (31, 32). CAL-27 cell line was grown in ATCC-formulated Dulbecco's Modified Eagle's Medium (DMEM), supplemented with 10% fetal bovine serum (FBS). SCC-25 and SCC-4 cell lines were grown in 1:1 mixture of DMEM and Ham's F12 medium containing 1.2 g/L sodium bicarbonate, 2.5 mM L-glutamine, 15 mM HEPES and 0.5 mM sodium pyruvate (DMEM:F12), supplemented with 400 ng/mL hydrocortisone and 10% FBS. SCC-4 cells were grown on ATCC 56-X.2™ feeder layer, MITC-STO cells, and the feeder cells (murine fibroblasts) were plated 24 hours before use at 2x10^6^/T75 in order to obtain a 30% confluent monolayer.

The mouse OSCC cell line MOC2 was used for the animal model of OSCC described in this study (33).

This cell line shows an aggressive growth phenotype (i.e., ability to form tumors with injection of as few as 10,000 cells) and is derived from a chemokine receptor CXCR3-deficient mouse on a pure C57BL/6 background (34). The MOC2 cell line was cultured in specific medium, following manufacturer's instructions. Briefly, for each Liter of medium, a 2:1 mixture of 626 mL Iscove's Modified Dulbecco's Medium (IMDM) and 313 mL Ham's nutrient mixture F10-F12, supplemented with 50 mL FBS and 10 mL Penicillin-Streptomycin was filtered in 1L filter flask. Finally, 1 mL of 5mg/mL Insulin, 800 μL of hydrocortisone solution, and 5 μL of Epidermal Growth Factor were added to the medium.

Detailed references of cell lines and reagents used in this study are reported in **Supplementary Table S3**.

### 2.3 Immunofluorescence, FACS analysis, and IFN-γ treatment

The cell surface expression of MHC-I, MHC-II, PD-1 and PD-L1 molecules was assessed by immunofluorescence and flow cytometry (BD FACSAria™ II Cell Sorter). Briefly, cells were washed twice with PBS, dissociated with trypsin-EDTA, and resuspended in complete medium. Cells were pelleted at 800 x *g* for 5 min, washed, the supernatant was discarded, and the pellet resuspended in PBS for FACS analysis. The following monoclonal antibodies were used as primary antibodies for the immune phenotype characterization of human OSCC cell lines (CAL-27, SCC-25, SCC-4): B9.12.1 anti-MHC-I (35) and D1–12 anti-MHC-II (DR) (36) used as hybridoma supernatants, followed by goat anti-mouse FITC, CD279 anti-PD-1 (clone EH12.2H7) PE, and CD274 anti-PD-L1 (clone 29E.2A3) PE, as previously described (**Supplementary Table S4**) (37, 38).

3x10^5^ OSCC cells were plated in 6 multi-well plates and treated with 500 U/mL of IFN-γ or with its vehicle. Seventy-two hours after treatment, the cells were collected and analyzed by immunofluorescence and flow cytometry, as indicated above. As positive and negative controls, RA and HepG2 cell lines were used, respectively. RA is a glioblastoma cell line that expresses MHC-I but not MHC-II molecules on cell surface. However, after *in vitro* stimulation with IFN-γ, cell surface MHC-II expression is induced, as described in previous experiments (39). Conversely, HepG2 is a hepatocarcinoma cell line whose MHC-II expression on cell surface remains negative after IFN-γ treatment, due to hypermethylation of the CIITA promoter IV (CIITA pIV) region, as previously demonstrated (38).

The cell surface expression of MHC-I (H2 MHC class I), MHC-II (I-A/I-E), PD-1 and PD-L1 molecules was assessed in murine MOC2 OSCC cell line by immunofluorescence and flow cytometry at baseline and 72 hours after IFN-γ treatment (1,000 U/mL), as previously described for human OSCC cell lines. The following antibodies were used for analysis: M1/42 anti-H2 MHC class I FITC, M5/114.15.2 anti-I-A/I-E PerCP Cyanine5.5, CD279 anti-PD1 (clone J43) PE, and CD274 anti-PD-L1 (clone MIH5) PE (**Supplementary Table S4**).

The data were analyzed by using FlowJo 9.5.2 software.

### 2.4 Animal model and study design

To test the efficacy of the MHC-II-based vaccination strategy in OSCC, we performed an *in vivo* experiment in a syngeneic mouse model (C57BL/6, H-2^b^ genotype, Charles River Laboratories Italia srl, Calco, Italy) (25). Briefly, we generated by genetic transfer stable MHC-II-expressing MOC2 tumor cells *in vitro* and injected them subcutaneously (s.c.) in mice to assess tumor rejection and/or tumor growth retardation compared with parental tumor cells. Following analyses included adoptive cell transfer and *in vivo* cell depletion experiments to better describe the role of CD4^+^ and CD8^+^ T lymphocytes in the immune response elicited against tumors.

Each experiment was repeated at least twice using five to eight mice per group.

All animal experiments were conducted according to relevant national and international guidelines and were approved by the local Animal Welfare Ethical Committee (OPBA) and by the Italian Ministry of Health (protocol n° 249/2022-PR).

### 2.5 Plasmid

CIITA cDNA was excised with EcoRI digestion from pcf-CIITA1–1130 and cloned by ligation into EcoRI-cleaved pAIP vector (Addgene), as previously described (40).

### 2.6 Generation of stable MHC-II-expressing MOC2 cell line (MOC2-CIITA)

MOC2 tumor cells were transfected with 3 μg of flag-CIITA (pAIP-fCIITA) expression vector or with pAIP empty vector (mock) by using FuGENE® HD Transfection kit (Promega, cat n° E2311), as previously described (41).

Both mock and CIITA-transfected MOC2 cells underwent puromycin selection at the concentration of 1.5 μg (Sigma Aldrich, cat n° P9620). For CIITA-transfected MOC2 cells, MHC-II-positive cells were enriched by fluorescence-activated cell sorting with a BD FACSAria™ II cell sorter and subjected to limiting-dilution cloning. The expression of the pAIP empty vector in MOC2-mock cells was assessed by RT-PCR by using the following primers to amplify Puromycin resistance cassette: forward, 5'-gcaacctccccttctacgagc-3'; reverse, 5'-gtgggcttgtactcggtcat-3'.

### 2.7 Vaccination and challenge

Syngeneic C57BL/6 female mice (H-2^b^) 7–8 weeks old were injected s.c. with 1x10^5^ cells/mL of either MOC2 CIITA-transfected tumor cells (MOC2-CIITA) or MOC2 parental tumor cells (MOC2-pc) or pAIP-transfected MOC2 cells (MOC2-mock). The expression of CIITA-driven MHC-II molecules was confirmed the day of the injection by immunofluorescence and flow cytometry, as described above.

Tumor growth along with the overall health condition of the mice were checked at least twice a week. The tumors were measured weekly using a caliper and registered in mm^2^.

The mice that did not show any tumor growth after injection with MOC2-CIITA were challenged with a s.c. injection of 1x10^5^ MOC2-pc and the tumor size was measured as above.

Each experiment was repeated at least twice using 5–8 mice per group.

### 2.8 *Ex-vivo* tumor analysis

Primary subcutaneous OSCC tumors were isolated and collected in DMEM 4 weeks after implantation. Mouse tumors were then diced into pieces measuring 2–4 mm. Tissue suspension was further broken down with mouse tumor dissociation kit (Miltenyi, cat n° 130–096-730) and gentleMACS™ Dissociator (Miltenyi, cat n° 130–093-235), following the manufacturer instructions.

### 2.9 Adoptive cell transfer

Spleens from challenged vaccinated mice that did not show parental tumor growth after 4 weeks from injection were harvested and processed. A single cell suspension was obtained and used to purify CD4^+^ and CD8^+^ T cells by CD4^+^ and CD8a^+^ Mouse T Cell Isolation Kit (Miltenyi Biotec GmbH, Cat n° 130–095-248/130–095-236, respectively). The purity of the selected cells was confirmed by flow cytometry using 145–2C11 anti-CD3e^+^ (BD, cat n° 553066), RM4–5 anti-CD4^+^ (BD, cat n° 550954), and 53–6.7 anti-CD8a^+^ (BioLegend, cat n° 100711) antibodies (25). At the same time, normal splenocytes were obtained from naïve C57BL/6 female mice of the same age.

Naïve female mice, 7–8 weeks old, were s.c. co-injected with 1x10^5^ MOC2-pc in a 100 μL volume of IMDM without FBS, along with either 1x10^7^ total immune splenocytes, CD4^+^ T cells, CD8^+^ T cells, or total naïve splenocytes as a control, in a tumor:immune cells ratios of 1:50, 1:15, 1:10, and 1:50, respectively.

The experiment was repeated at least twice using 5–8 mice per group. The mice under experimentation were followed for 4 weeks and the data were recorded as described before.

### 2.10 *In vivo* cell depletion

Eight-week old mice were injected intraperitoneally (i.p.) -9, -7, -5, -2, -1 days prior to tumor injection (day 0) and +2, +5, +7 days after, with 100 μL/injection of anti-CD4^+^ (aCD4 GK 1.5) or anti-CD8^+^ (aCD8 2.43.5) antibodies, or with control rat polyclonal Ig (24). For CD4^+^ depletion we performed an additional injection at day -12. To verify the efficacy of the procedure before tumor injection, CD4^+^ and CD8^+^ T cells depletion was tested by immunofluorescence and flow cytometry on splenocytes derived from treated mice, incubated with anti-mouse CD4^+^ and anti-mouse CD8^+^ antibodies, and compared with splenocytes isolated from untreated mice.

Treated and untreated mice were injected s.c. with either 1x10^5^ MOC2-CIITA or MOC2-pc at day 0. The size of the tumor was measured weekly, as described before.

### 2.11 Statistical analysis

The hypothesis of independence between categorical variables was tested by Chi-square test or Mann-Whitney test. Variables were considered dependent when p ≤ 0.05.

The other analyses were performed using GraphPad Prism 10 (GraphPad Software, http://www.graphpad.com), and the multiple unpaired Student's t-test was conducted to determine significance.

Comparisons between groups were considered statistically significant when the corresponding p-value was ≤ 0.05.

## 3 Results

### 3.1 Recruited population and histopathological assessment

The 23 patients affected by OSCC were 13 males and 10 females, with a mean age of 72.52 ± 12 years, without differences between the sexes (p=0.217). All the included samples were HPV-negative, conventional-type OSCC (**Table 1A**).

**Table 1:** Epidemiological profile of oral cancer patients (A) and correlations between immune cell infiltration and clinical outcomes (B).

A. Population characteristics and disease staging.				
	*Males*	*Females*	*Total*	
*N*	13	10	23	
*Mean age*	69.77 ± 11.08	76.1 ± 12.78	72.52 ± 12.0	p=0.217
*Disease stage*				p=0.303
I	3	3	6	
II	6	1	7	
III	2	3	5	
IV	2	3	5	

B. Correlations between immune cell infiltration and clinical outcomes.				
	*LN metastasis^a^*		*5-year CRD^b^*	
*N*	7/23		6/23	
*Intratumor infiltrate*		p=0.530		p=0.343
0	5		3	
1+	1		3	
2+	1		0	
*Stromal infiltrate*		p=0.533		p=0.666
0	0		0	
1+	2		2	
2+	5		4	

^a^cervical lymph node metastasis at diagnosis (LN metastasis).

^b^cancer-related death (CRD).

Based on AJCC and WHO updated diagnostic criteria, the oral cancer samples analyzed were at different stage of tumor progression. Seven patients showed LN metastasis at diagnosis, while six died within 5 years for cancer-related disease (CRD). Among deceased patients, three did not show LN metastasis at diagnosis and developed recurrent disease after primary treatment.

Based on the presence of immune cellular infiltrate in tumor epithelial islands and stroma, tumors were classified as 0, 1+, and 2+ (see Materials and Methods). The degree of cellular infiltration in tumor epithelial nests did not correlate with either LN metastasis at diagnosis (p=0.530) or 5-year CRD (p=0.343) (**Table 1B**). A similar trend was observed in relation to the degree of inflammation in the stroma (LN metastasis p=0.533; 5-year CRD p=0.666).

### 3.2 MHC-II but not MHC-I expression on tumor cells correlates with CD4^+^ and CD8^+^ T-lymphocyte infiltration in tumor tissue

The expression of both MHC-I and MHC-II molecules was assessed on OSCC tumor tissues by IHC, as stated in Materials and Methods.

MHC-I antigens were detectable on tumor cells in all 22 analyzed samples; one sample was not evaluable due to exhaustion of tumor area. However, the expression of MHC-I staining was variable, with 7, 7, and 8 patients showing low, moderate, and high degree of expression, respectively (**Table 2**). The level of MHC-I expression did not statistically correlate with clinical outcomes, even though low MHC-I expression levels were associated with a higher incidence of LN metastasis at diagnosis and more advanced stage of disease. Indeed, in patients with stage IV oral cancer MHC-I molecules were significantly hypoexpressed on tumor cells (p=0.025, **Table 2**).

**Table 2:** Correlations between MHC-I and MHC-II molecule expression on tumor cells and clinical outcomes.

	MHC-I expression					MHC-II expression			
	*Low*	*Moderate*	*High*	Total	p-value	*Negative*	*Positive*	Total	p-value
*N*	7	7	8	22		13	10	23	
*Gender M/F*	5/2	4/3	4/4	13/9	0.696	10/3	3/7	13/10	**0.024**
*Mean age*	66.71 ± 12.12	76.29 ± 12.55	73.38 ± 11.61	72.18 ± 12.17	0.334	68.69 ± 11.71	77.5 ± 10.97	72.52 ± 12.0	0.08
*LN metastasis^a^*	2	4	1	7	0.176	2	5	7	0.074
*5-year CRD^b^*	0	3	3	6	0.142	3	3	6	0.708
*Stage*					0.138^c^				0.842
I	1	2	2	5		4	2	6	
II	3	0	4	7		4	3	7	
III	2	1	2	5		3	2	5	
IV	1	4	0	5		2	3	5	

^a^cervical lymph node metastasis at diagnosis (LN metastasis).

^b^cancer-related death (CRD).

^c^MHC-I molecules were significantly hypoexpressed in stage IV oral cancer (p=0.025).

Statistically significant results are highlighted in bolded character.

MHC-II-positive tumor cells were found in 43.5% of the recruited patients (10 out of 23), particularly in females (p=0.024, **Table 2**). As observed for MHC-I, MHC-II expression level on tumor cells did not correlate with either LN metastasis at diagnosis (p=0.074) or 5-year CRD (p=0.708, **Table 2**).

We then assessed whether a correlation existed between MHC molecules expression on tumor cells and the presence of specific immune cell infiltration in tumor epithelial nests and stroma (**Table 3**). We did not find statistically significant correlations between MHC-I molecule expression and tumor epithelial nest infiltration, considering both total immune infiltrate (p=0.263) and specific cell subpopulations (CD4^+^ p=0.325; CD8^+^ p=0.970; CD68^+^ p=0.137). Similar findings were observed in the stroma (total immune cells p=0.575; CD4^+^ p=0.911; CD8^+^ p=0.537; CD68^+^ p=0.911) (**Table 3**).

**Table 3:** Correlations between MHC antigens expressed on tumor cells and infiltration of both total and specific cell subpopulations in tumor epithelial tissues and stroma.

	MHC-I expression			Total	p-value	MHC-II expression		Total	p-value
	*Low*	*Medium*	*High*			*Negative*	*Positive*		
N	7	7	8	22		13	10	23	
Tumor epithelial nests									
*Total infiltrate*					0.263				0.097
0	5	4	4	13		8	5	13	
1+	0	3	3	6		5	2	7	
2+	2	0	1	3		0	3	3	
*CD4^+^ TH*					0.325				**0.047**
Low	6	5	4	15		11	4	15	
High	1	2	4	7		2	5	7	
*CD8^+^ CTL*					0.970				**0.012**
Low	3	3	3	9		8	1	9	
High	4	4	5	14		5	9	14	
*CD68^+^ cells*					0.137				0.099
Low	5	2	2	9		7	2	9	
High	2	5	6	13		6	8	14	
Stroma									
*Total infiltrate*					0.575				**0.026**
0	3	2	3	8		6	2	8	
1+	1	3	4	8		6	2	8	
2+	3	2	1	6		1	6	7	
*CD4^+^ TH*					0.911				**0.026**
Low	5	5	5	15		11	4	15	
High	2	2	3	7		2	6	8	
*CD8^+^ CTL*					0.537				0.072
Low	5	3	5	13		10	4	14	
High	2	4	3	9		3	6	9	
*CD68^+^ cells*					0.911				0.062
Low	2	2	3	7		6	1	7	
High	5	5	5	15		7	9	16	

Statistically significant results are highlighted in bolded character.

**Figure 1:**
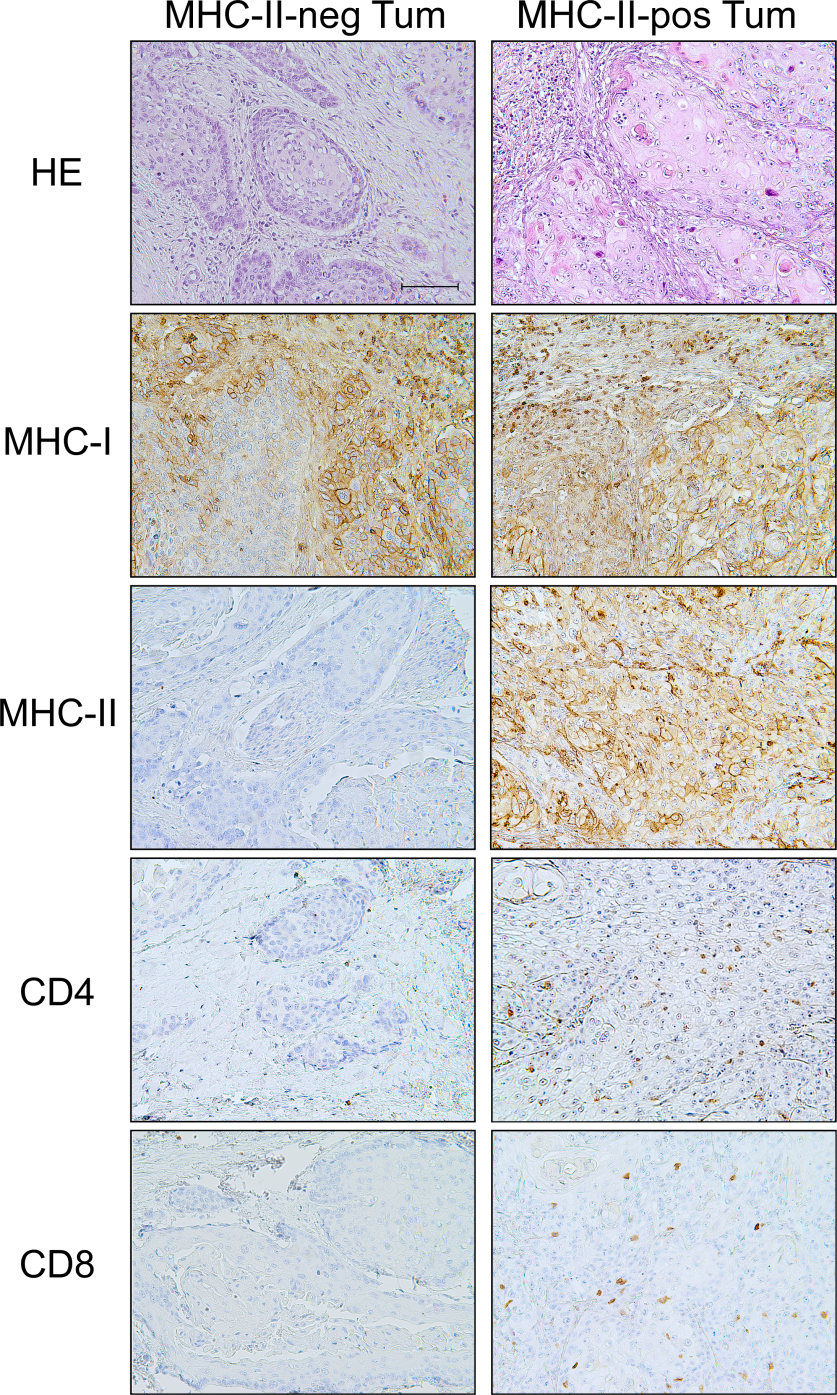
MHC-II-positive OSCC tumors are "hot" tumors with specific infiltration of both CD4^+^ TH and CD8^+^ CTL with respect to the "cold" MHC-II-negative tumors. Haematoxylin-eosin (HE) staining of two examples of MHC-II-negative and MHC-II-positive tumors (HE). The tumor tissue of the MHC-II-positive OSCC showed infiltration by immune cells, mainly lymphocytes. IHC staining of MHC-I antigens revealed that tumor cells in both MHC-II-negative and MHC-II-positive tumors expressed MHC-I molecules, with variable degree of intensity, i.e., moderate and high expression, respectively (MHC-I). On the other hand, IHC staining for MHC-II antigens clearly showed the absence of MHC-II expression in MHC-II-negative OSCC and a high expression of these molecules on the cell surface of MHC-II-positive OSCC (MHC-II). A significant infiltration of CD4^+^ TH cells was detected in tumor tissues of the MHC-II-positive OSCC, as opposed to MHC-II-negative OSCC (CD4). Similarly, the infiltration of CD8^+^ CTL cells was detected in MHC-II-positive OSCC tumor tissues, but not in MHC-II-negative OSCC (CD8). (200X original magnification, scale bar 100 μm).

Interestingly, although MHC-II expression did not statistically correlate with the total immune cell infiltrate in tumor islands (p=0.097), it strongly correlated with a high presence of both CD4^+^ TH (p=0.047) and CD8^+^ CTL (p=0.012), but not with CD68^+^ cells (p=0.099) (**Table 3**, **Figure 1**). As for the stroma, MHC-II expression on tumor cells was associated with the presence of high total immune cells (p=0.026) and high CD4^+^ TH (p=0.026), but not with CD8^+^ CTL (p=0.072) and CD68^+^ cells (p=0.062) (**Table 3**).

### 3.3 PD-L1 molecule is co-expressed with MHC-II molecules on OSCC tumor cell surface

To further characterize the expression of immune markers of relevance for immunotherapy approaches, we analyzed the presence of immune checkpoint PD-1 and its major ligand PD-L1.

PD-1 was expressed on the surface of immune cells intermingled among tumor epithelial cells and infiltrating the stroma. On the other hand, PD-L1 was present on both immune and tumor cells. In particular, 11/23 (i.e., 47.8%) tumors showed PD-L1 expression on tumor cells (TPS mean: 49.09 ± 24.06%) whereas CPS score, which includes PD-L1 expression both in tumor and immune cells, was positive in almost all cases (i.e., 19/20), with high expression detected in 13/19 patients (CPS mean: 40.3 ± 34.84).

We next investigated a possible correlation between the expression of the immune checkpoints on immune cells (PD-1) and tumor cells (PD-L1) and the clinical outcomes (**Table 4A**). The expression of these molecules did not correlate with clinical outcomes, except for PD-1-positive immune cells in tumor nests that correlated with the presence of LN metastasis at diagnosis (p=0.015) (**Table 4A**).

**Table 4:** Correlations between immune checkpoints (PD-1; PD-L1) and clinical outcomes (A), MHC antigens (I and II) expression on tumor cells (B), and specific immune cell infiltration (C).

		PD-1				PD-L1					
		High Intraepithelial PD-1		High Stromal PD-1		High PD-L1 CPS^a^		PD-L1-positive Tumor cells^b^		High Stromal PD-L1	
		N	p-value	N	p-value	N	p-value	N	p-value	N	p-value
A	*Clinical outcomes*										
	LN metastasis^c^: present absent	5/7 3/16	**0.015**	5/7 10/16	0.679	5/7 8/12	0.829	4/7 7/16	0.554	4/7 6/16	0.382
	5-year CRD^d^ Alive (or non-cancer death)	3/6 5/17	0.363	2/6 13/17	0.06	3/4 10/15	0.750	3/6 8/17	0.901	2/6 8/17	0.560
B	*MHC antigens (tumor cells)*										
	MHC-I Low Moderate High	3/7 2/7 3/8	0.854	5/7 4/7 5/8	0.854	3/5 4/6 5/7	0.918	2/7 3/7 5/8	0.415	3/7 3/7 3/8	0.970
	MHC-II Positive Negative	5/10 3/13	0.179	9**/**10 6/13	**0.029**	9/10 4/9	**0.033**	9/10 2/13	**< 0.001**	5/10 5/13	0.580
C	*Immune cells*										
	Total immune cells: 0 1+ 2+	3/13 3/7 2/3	0.312	- 4/8 11/15	0.263					- 1/8 9/15	**0.029**
	CD4^+^ TH Low High	6/15 2/7	0.604	9/15 6/8	0.472					5/15 5/8	0.179
	CD8^+^ CTL Low High	2/15 6/8	**0.003**	7/14 8/9	**0.05**					5/14 5/9	0.349
	CD68^+^ cells Low High	3/9 5/14	0.907	4/7 11/16	0.591					2/7 8/16	0.340

^a^Combined Positive Score (CPS): [(PD-L1-positive tumor cells + PD-L1-positive mononuclear inflammatory cells)/total number of viable cells)] x 100.

^b^Tumor Proportion Score (TPS) ≥ 10.

^c^cervical lymph node metastasis at diagnosis (LN metastasis).

^d^cancer-related death (CRD).

Statistically significant results are highlighted in bolded character.

We next verified whether the expression of MHC-I and/or MHC-II molecules on tumor cells was associated to the presence of immune checkpoints (**Table 4B**). While there was a higher number of PD-1-positive immune cells in the stroma of MHC-II-positive tumors (p=0.029), this correlation did not exist with MHC-I (p=0.854).

**Figure 2:**
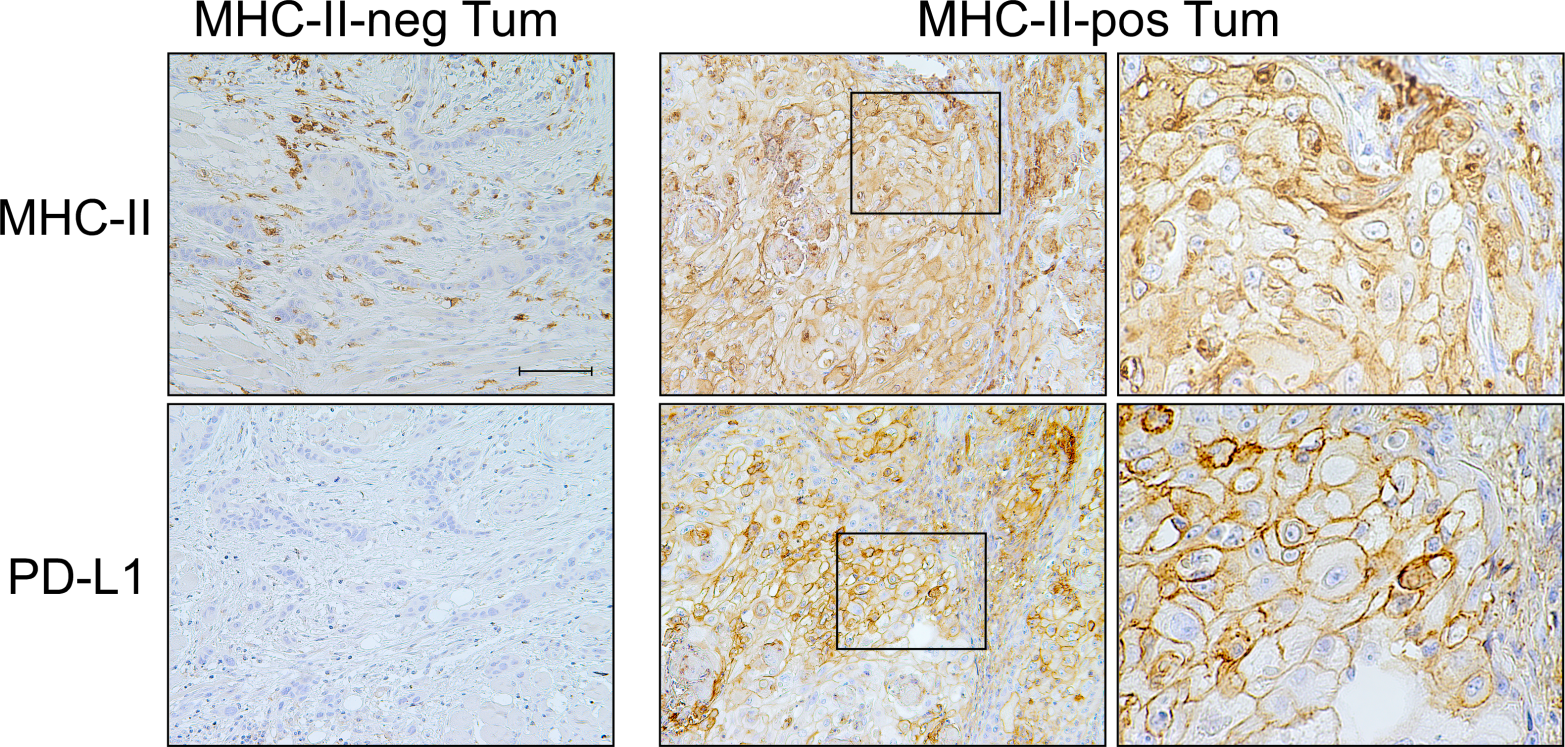
PD-L1 checkpoint molecule is co-expressed with MHC-II-antigens on OSCC tumor cells. Representative MHC-II-negative OSCC in which tumor cells do not express PD-L1 molecule on their surface (MHC-II-neg Tum panel column, 200X, scale bar 100 μm). MHC-II-positive OSCC tumor cells highly express PD-L1 (MHC-II-pos Tum panel column, left, 200X). At higher magnification, the detection of both MHC-II antigens and PD-L1 on the cell membrane of tumor cells became clearly appreciable (MHC-II-pos Tum panel column, right, 600X).

Similarly, the CPS was higher in MHC-II-positive tumors (p=0.033) and was independent from MHC-I antigen expression (p=0.918). At variance with PD-1, PD-L1 expression on stromal cells did not correlate either with MHC-I (p=0.970) or MHC-II molecules (p=0.580). Interestingly, however, most of the PD-L1-positive tumors expressed a high level of MHC-II molecules (p < 0.001) (**Table 4B**, **Figure 2**).

Finally, we correlated the expression of both PD-1 and PD-L1 and the different immune cell subpopulations infiltrating the tumor epithelial nests or present in the stroma (**Table 4C**). High detection of PD-1 in immune cells infiltrating tumor epithelial islands correlated with the presence of high expression of CD8^+^ CTL (p=0.003), but not with CD4^+^ TH and CD68+ cells (p=0.604 and p=0.907, respectively). Similar findings were observed for the stroma (CD8^+^ CTL p=0.05; CD4^+^ TH p=0.472; CD68^+^ cells p=0.591) (**Table 4C**).

A statistically significant correlation between PD-L1 expression and high total immune cells infiltrating the stroma (p=0.029) was observed, although it was not related to specific T cell subpopulation (**Table 4C**).

### 3.4 MHC-II molecules are variably expressed on human OSCC cell lines and can be rescued by IFN-γ

**Figure 3:**
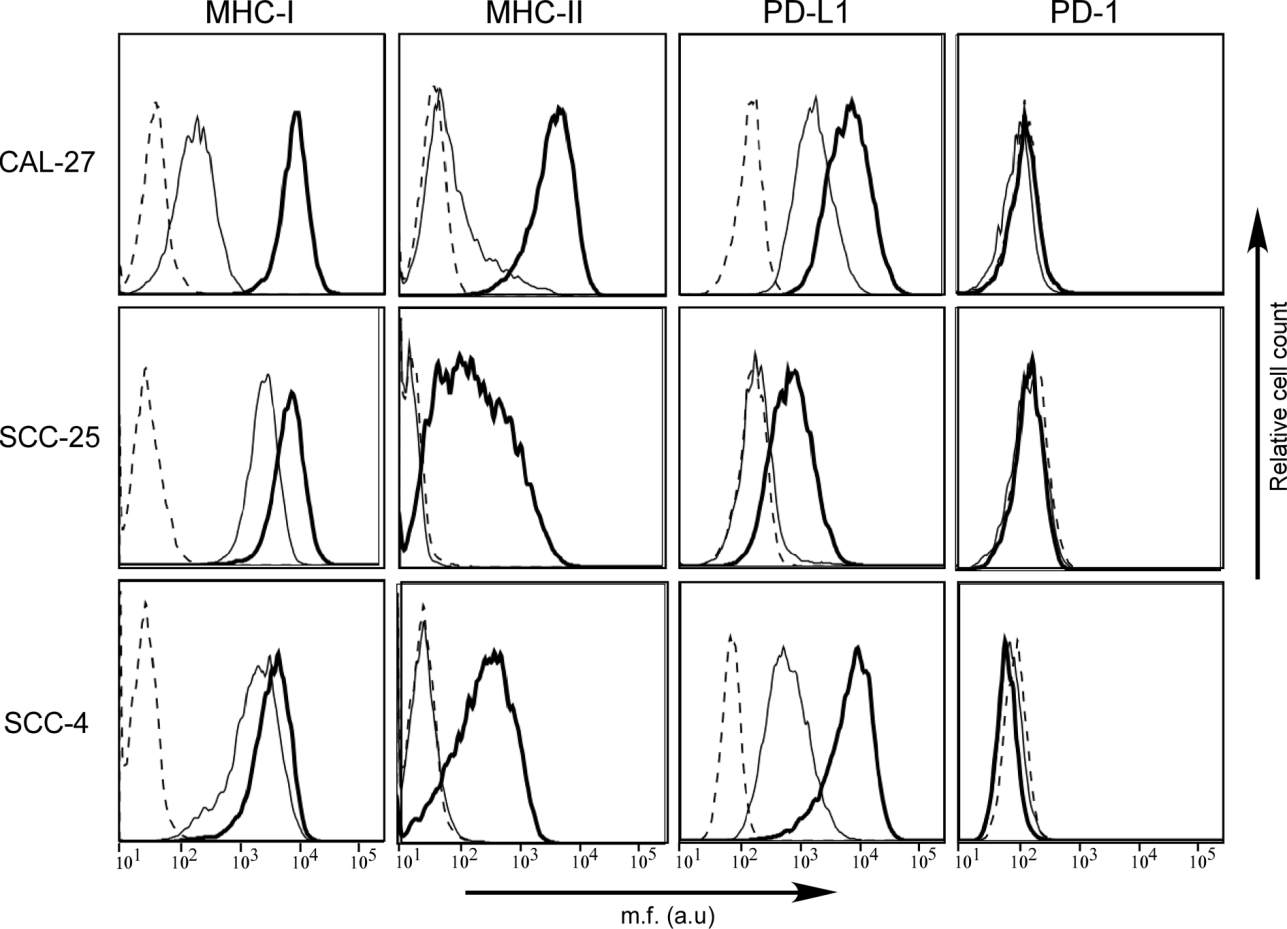
MHC-II antigens but also PD-L1 checkpoint molecules are expressed in human OSCC cell lines after IFN-γ treatment. MHC-I, MHC-II, PD-1, and PD-L1 cell surface phenotype analysis of CAL-27, SCC-25, and SCC-4 human oral cancer cell lines was carried out by immunofluorescence and FACS analysis. The first two columns of histograms represent fluorescence profiles of the cells indicated on the left incubated with specific anti-MHC-I and anti-MHC-II (HLA-DR) antibodies followed by incubation with FITC-conjugated anti-mouse antibody as a second reagent. Similarly, the third and fourth columns of histograms display fluorescence phenotype of the cells incubated with specific anti-PD-L1 and anti-PD-1 antibodies, respectively, followed by incubation with PE-conjugated matched isotype control antibody as a second reagent. Cells were either untreated (solid line) or treated with IFN-γ (bold line). Controls (dashed line) were cells incubated with the second reagent only. Mean fluorescence (m.f.) values are expressed in the abscissa as arbitrary units (a.u.).

In order to investigate similarities or differences in the cell surface phenotype between OSCC in tumor tissues and in isolated OSCC cell lines, we studied three human OSCC cell lines, namely CAL-27, SCC-25, and SCC-4 (**Figure 3**).

As far as the MHC cell surface expression, we found that MHC-I molecules were expressed in all three cell lines, although at different levels, mimicking what we observed on tumor tissues. In particular, the MHC-I expression detected on CAL-27 cell line was significantly lower when compared to that of SCC-25 and SCC-4. On the contrary, MHC-II molecules were slightly expressed only on the surface of CAL-27 cells (**Figure 3**). Again, this result resembled what we observed in human tissues, with a subgroup of patients showing MHC-II-positive tumors.

The expression of MHC genes could be rescued/increased by treatment with the inflammatory cytokine IFN-γ. As far as MHC-I expression, it increased to reach similar levels in all three cell lines after treatment with the cytokine. This was particularly relevant for CAL-27 (**Figure 3**, bold line). As far as MHC-II, it should be underlined that the IFN-γ-dependent rescuing of MHC-II gene expression is mediated by a direct action on the expression of CIITA, the MHC class II transactivator, which in turn activates the expression of MHC-II genes (20). MHC-II expression could be rescued in all three cell lines although to different extent (**Figure 3**, bold line), and this correlated to *de novo* CIITA expression (**Supplementary Figure S1**).

We next investigated the expression of PD-1 and PD-L1 in the three OSCC cell lines before and after treatment with IFN-γ. As expected, the PD-1 molecule, which is usually expressed by immune cells, was not detected on the cell surface of the OSCC cell lines, neither before nor after treatment with IFN-γ (**Figure 3**).

In contrast, as we observed in histopathological samples, PD-L1 was differently expressed by the three OSCC cell lines, with CAL-27 and SCC-4 clearly expressing the marker and SCC-25 not in normal cell culture condition. After treatment with IFN-γ, PD-L1 expression was rescued in SCC-25 and upregulated in the other two OSCC cell lines (**Figure 3**, bold line).

### 3.5 Establishing an animal model to assess the potential of immune vaccination and immunotherapy against OSCC

Our previous investigations have established the strong potential of an immune vaccination against tumors by using cellular tumor models of various histotype in mice (24, 25, 41). The rational was to render tumor cells MHC-II-positive by genetic transfer of CIITA and then assess the *in vivo* behavior of CIITA-tumor cells. Given the importance of oral squamous cell carcinoma both as incidence and poor therapeutic armamentarium in human, and the rather scanty availability of experimental systems to assess innovative therapies, we wanted to test whether murine OSCC tumor cells genetically modified to express constitutively MHC-II molecules could be recognized and rejected and/or delayed in their growth when injected into syngeneic immunocompetent recipients.

**Figure 4:**
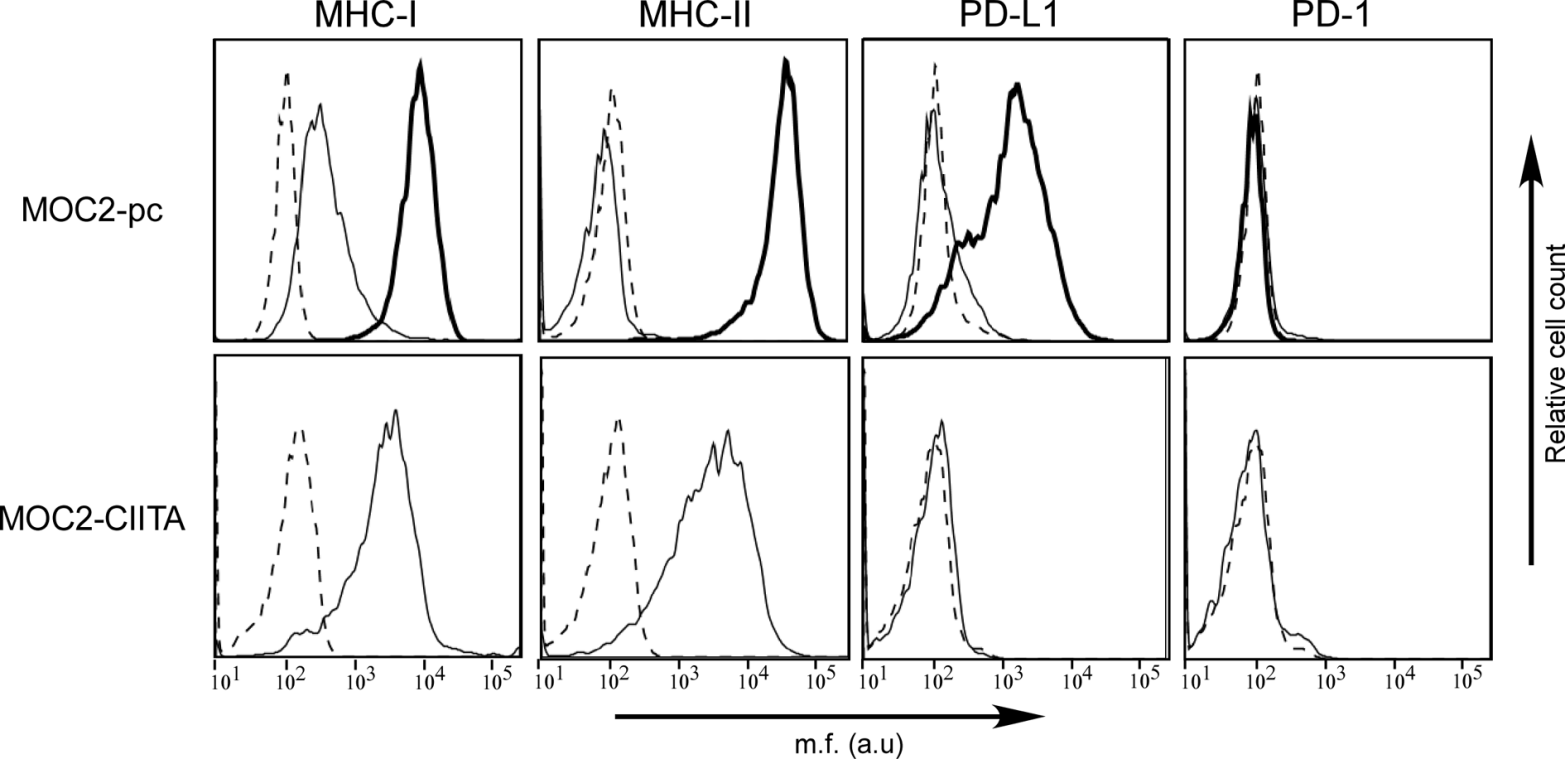
The phenotype of OSCC MOC2-pc cell line after IFN-γ treatment mirrors the phenotype of human OSCC cell lines, but PD-L1 is not expressed in MOC2-CIITA cells. MHC-I, MHC-II, PD-1, and PD-L1 cell surface phenotype analysis of MOC2 murine oral cancer cell line was carried out by immunofluorescence and FACS analysis. Histograms represent fluorescence profile of the cells indicated on the left incubated with specific anti-MHC-I (H2 MHC class I), anti-MHC-II (I-A/I-E), anti-PD-L1 and anti-PD-1 antibodies, respectively, followed by incubation with specific antibodies as a second reagent. MOC2-pc: MOC2 parental cells (MOC2-pc) immune phenotype reflected results obtained in human OSCC cell lines, i.e., IFN-γ treatment upregulated MHC-I antigens expression and induced the expression of both MHC-II and PD-L1 molecules. Cells were either untreated (solid line) or treated with IFN-γ (bold line). Controls (dashed line) were cells incubated with the second reagent only. MOC2-CIITA: MOC2 tumor cells transfected with CIITA revealed a constitutive expression of MHC-I and MHC-II antigens, while the PD-L1 molecules expression was not induced. Transfected cells were indicated with solid line, while controls (i.e., cells incubated with the second reagent only) with dashed line. Mean fluorescence (m.f.) values are expressed in the abscissa as arbitrary units (a.u.).

The murine OSCC cell line MOC2 of H-2^b^ genetic background was first characterized for the cell surface expression of MHC-I and MHC-II, as well as PD-1 and PD-L1. MHC-I antigens were expressed on MOC2 cell surface, while MHC-II molecules were not detectable (**Figure 4**, MOC2-pc). However, as observed in OSCC human cell lines, IFN-γ treatment upregulated MHC-I expression and induced MHC-II molecules on the cell surface of MOC2 cell line (**Figure 4**, MOC2-pc, bold line). The expression of MHC-II molecules was driven by the induction of CIITA gene transcription upon IFN-γ treatment (**Supplementary Figure S2**).

As far as the immune checkpoints, PD-1 was not detected either before or after treatment with the cytokine. Differently from what we could observed in OSCC human cell lines, MOC2 cell line did not express PD-L1 molecule (**Figure 4**, MOC2-pc). However, expression of PD-L1 was clearly induced after treatment with IFN-γ, consistent with what we observed in human cell lines (**Figure 4**, MOC2-pc, bold line).

MOC2 cell line was selected to be transfected with CIITA and used in *in vivo* experiments. A transfectant stably expressing CIITA-driven MHC-II cell surface molecules (MOC2-CIITA) was selected by cell sorting. **Figure 4** (MOC2-CIITA) shows the MHC-II cell surface expression of MOC2-CIITA compared to the parental untransfected control (MOC2-pc). Of note, MOC2-CIITA transfected cell line did not express PD-L1, differently from results obtained in parental cell line after treatment with IFN-γ (**Figure 4**, MOC2-pc, bold line). It should be emphasized that stable expression of CIITA in MOC2 tumor cells did not affect their growth rate *in vitro* (**Supplementary Figure S3**).

**Figure 5:**
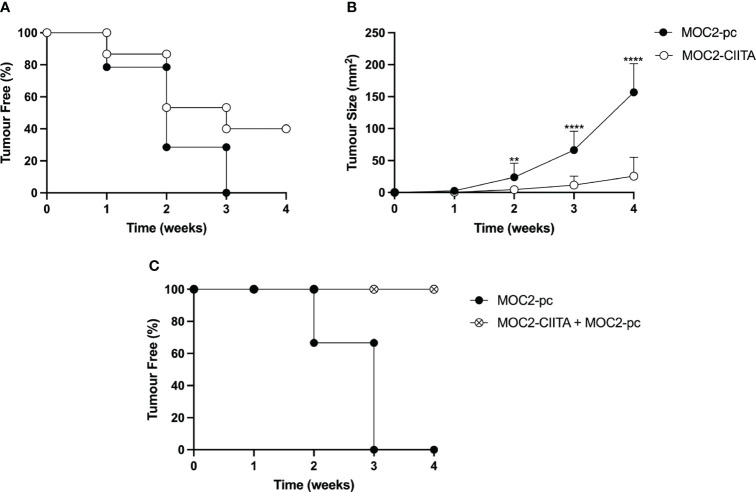
*In vivo* vaccination with MHC-II-positive OSCC tumor cells (MOC2-CIITA) elicits a strong anti-tumor immune response capable of tumor rejection or growth retardation, and resistant to challenge with parental OSCC tumor cells (MOC2-pc). MOC2-CIITA tumors were rejected or strongly retarded in their growth after s.c injection in C57BL/6 mice (see Materials and Methods). **(A)** The Kaplan-Meyer curve shows that 40% of mice that were vaccinated with MOC2-CIITA (empty circles) did not develop cancer after 4 weeks, while all animals injected with MOC2-pc parental tumor (full circles) showed tumor development within 3 weeks. Mice were followed for tumor take (ordinate: percent of tumor-free mice) over time (abscissa). **(B)** MOC2-CIITA (empty circles) and MOC2-pc (full circles) growing tumors were measured for their tumor size (ordinate) over time (abscissa). p-values were obtained via unpaired Student t-test (**p < 0.01; ****p < 0.0001). **(C)** Animals vaccinated with MOC2-CIITA that did not develop cancer (see panel A) were challenged with MOC2-pc. The Kaplan-Meyer curve shows that 100% of challenged vaccinated animals (crossed open circles) did not develop cancer, while naïve animals injected with MOC2-pc (full circles) showed tumor development within 3 weeks. Mice were followed for tumor take (ordinate: percent of tumor-free mice) over time (abscissa).

MOC2-CIITA cells were s.c. injected into naïve syngeneic C57BL/6 mice and tumor growth was monitored over time, as previously described. At 4 weeks after tumor injection, MOC2-CIITA tumors were rejected in 40% of mice with respect to their MOC2-pc counterpart (**Figure 5A**). Importantly, in the remaining 60% of MOC2-CIITA injected mice the kinetics of tumor growth was strongly delayed and after four weeks the average tumor size was seven-fold lower (p<0.0001) compared to parental MOC2 tumors (**Figure 5B**). Notably, similar findings were obtained when comparing the tumor growth of MOC2-CIITA cells with MOC2 cells stably transfected with pAIP empty vector (MOC2-mock). The expression of pAIP empty vector was assessed by RT-PCR for the presence of Puromycin cassette in MOC2-mock cells compared to MOC2 parental cells (**Supplementary Figure S4A**, compare lane 2 and 3). Indeed, 50% of MOC2-CIITA injected mice rejected the tumor (**Supplementary Figure S4B**) and the remaining 50% showed a significant delay in tumor growth compared to MOC2-mock tumor-bearing mice (**Supplementary Figure S4C**). At four weeks the MOC2-CIITA tumor size was seven-fold lower compared to MOC2-mock tumors (p<0.0001).

To investigate whether the rejection of CIITA-tumors in mice was attributable to the generation of an adaptive immune response that could protect the animals from a challenge with untransfected parental (pc) tumors, MOC2-CIITA tumor-free mice were challenged with MOC2-pc. Results clearly showed that animals immunized and protected from CIITA-tumors fully rejected, in 100% of the cases, the untransfected MHC-II-negative parental tumors (**Figure 5C**).

**Figure 6:**
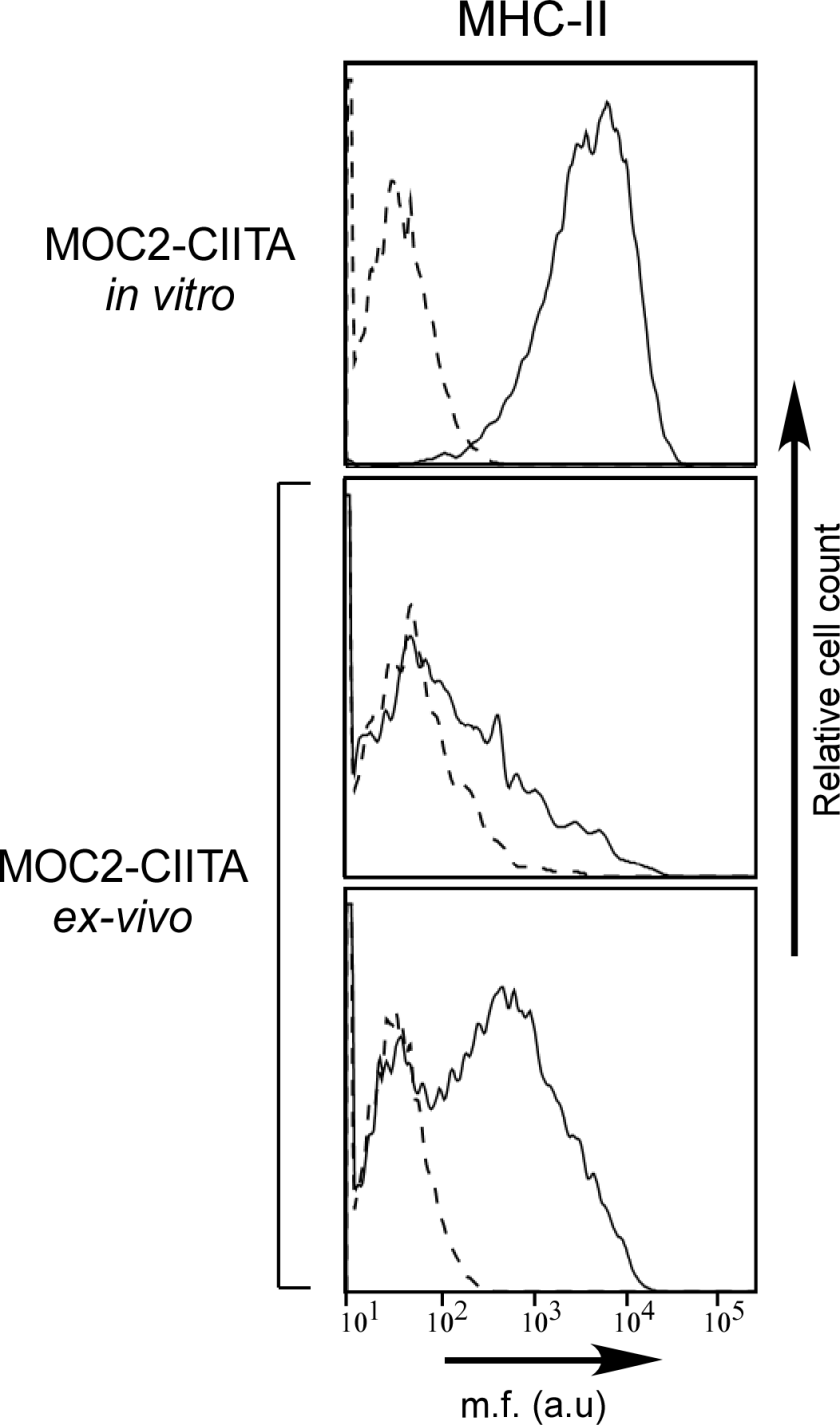
MHC-II expression is remarkably reduced in MOC2-CIITA tumors explanted 4 weeks after injection. MOC2-CIITA tumors were explanted at 4 weeks after injection and tumor cells were isolated as described in Materials and Methods (*ex-vivo*). MHC-II expression (solid line) was evaluated by immunofluorescence and FACS analysis in MOC2-CIITA cells the day of injection (MOC2-CIITA *in vitro*) and in tumor cells isolated from explanted tumors after 4 weeks from injection (MOC2-CIITA *ex-vivo*). Representative analysis of two MOC2-CIITA bearing mice are shown. Controls (i.e., cells incubated with the second reagent only) were indicated with dashed line. Mean fluorescence (m.f.) values are expressed in the abscissa as arbitrary units (a.u.).

Remarkably, in mice in which MOC2-CIITA tumors were delayed in their growth *in vivo*, the expression of MHC-II molecules was significantly reduced compared to that of MOC2-CIITA cells the day of injection, as demonstrated by FACS analysis of tumor cells isolated from MOC2-CIITA tumors explanted at 4 weeks after injection (**Figure 6**).

From these results, we conclude that MOC2 tumor cells of aggressive behavior *in vivo* not only can be rejected or strongly delayed in their growth after *de novo* expression of CIITA-mediated MHC-II molecules, but also induce an immunological memory capable to confer resistance to challenge with MHC-II-negative parental tumors.

To assess whether protection from tumor growth after vaccination with MOC2-CIITA was associated to a change in the phenotype of T cells with respect to tumor-bearing mice, we have analyzed the spleen cells from animals bearing the MOC2 parental tumor as compared to animals protected by vaccination with MOC2-CIITA. **Supplementary Figure S5** shows that MOC2 tumor-bearing mice display a preferential TH2 phenotype, as demonstrated by an increased secretion of IL-4, particularly when stimulated *in vitro* with MOC2 parental or MOC2-CIITA cells, while MOC2-CIITA vaccinated mice express a preferential TH1 phenotype when stimulated *in vitro* with either parental MOC2 or, more importantly, with MOC2-CIITA cells. These results suggest a preferential triggering of TH1 responses following vaccination with CIITA-modified tumor cells.

**Figure 7:**
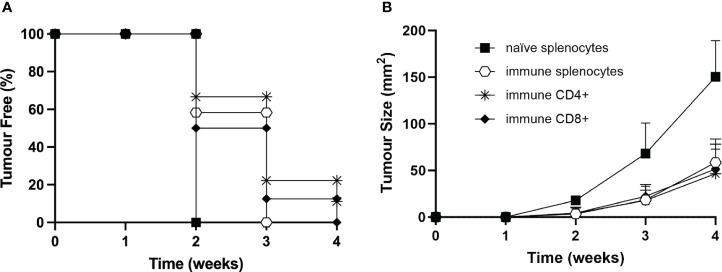
Adoptive cell transfer of immune CD4^+^ and/or CD8^+^ T lymphocytes from vaccinated to naïve animals confers resistance to tumor development or strongly reduces tumor growth rate. **(A)** Total splenocytes (open hexagons), CD4^+^ T cells (asterisks), CD8^+^ T cells (full rhombuses) of MOC2-CIITA vaccinated mice fully rejecting the tumor (immune), or naïve splenocytes (full squares) were co-injected with parental MOC2-pc tumor cells into naïve recipients and tumor growth was followed over time. Kaplan-Meyer curve shows results in terms of observed percentage of tumor-free mice (ordinate) over time (abscissa). **(B)** The size of tumors developing in mice receiving adoptive cell transfer of immune splenocytes (open hexagons), CD4^+^ T cells (asterisks), CD8^+^ T cells (full rhombuses) as described in **(A)** or naïve splenocytes was measured over time (abscissa) and reported in square millimeters (mm^2^) in the ordinate. p-values were obtained via multiple unpaired Student t-test for total immune splenocytes (week 2: p < 0.0001, week 3: p < 0.001, week 4: p < 0.0001), purified immune CD4^+^ splenocytes (week 2: p < 0.001, week 3: p < 0.01, week 4: p < 0.001) and purified immune CD8^+^ splenocytes (week 2: p < 0.001, week 3: p < 0.01, week 4: p < 0.0001), as compared to naïve splenocytes.

To define more precisely the contribution of the immune cell subpopulations involved in the triggering and maintenance of the protective antitumor response after vaccination with MOC2-CIITA cells, experiments of adoptive cell transfer (ACT) of immune splenocytes were performed (**Figure 7**). Total spleen cells, purified CD4^+^ and CD8^+^ T cells derived from mice that had rejected MOC2-CIITA and further rejected MOC2-pc after challenge, were selectively co-injected with MOC2-pc into naïve C57BL/6 mice. The ratio of tumor cells versus total splenocytes, CD4^+^ and CD8^+^ T cells was 1:50, 1:15, and 1:10 respectively, to mimic the relative proportion of the various lymphocyte subpopulations of the spleen. Tumor growth in the distinct groups of mice was followed for 4 weeks.

Importantly, immune CD4^+^ T cells were able to strongly reduce MOC2-pc tumor growth in 89% of the animals and confer tumor protection in the remaining 11% of mice (**Figure 7**). Virtually superimposable results were obtained by ACT with immune CD8^+^ T cells. Interestingly, the protective effect of immune CD4^+^ and CD8^+^ T cells was comparable to the effect obtained with total immune spleen cells, further reiterating the importance of both T cell subpopulations in the anti-tumor response (**Figure 7**).

**Figure 8:**
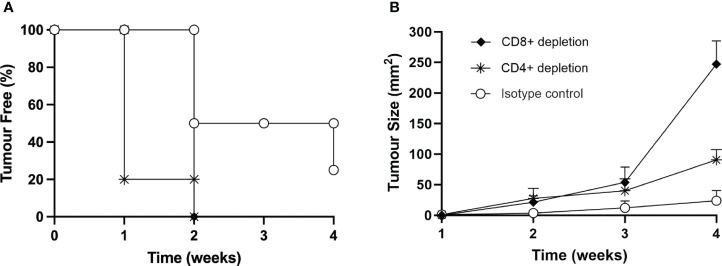
*In vivo* depletion of both CD4^+^ or CD8^+^ T cells nullifies the immune response elicited by the vaccination with MOC2-CIITA cells. Naïve animals were treated with anti-CD4^+^ (asterisks), anti-CD8^+^ (full rhombuses) or control (open circles) monoclonal antibodies as described in the text and then injected with MOC2-CIITA tumor cells. **(A)** The Kaplan-Meyer curve shows the percentage of tumor-free mice after receiving the above treatments (ordinate) followed over time (abscissa). **(B)** Shown in the figure is the size of tumors, measured in square millimeters (mm^2^) (ordinate), developing over time (abscissa) in mice treated with anti-CD4^+^ (asterisks), anti-CD8^+^ (full rhombuses) or control (open circles) monoclonal antibodies. p-values were obtained via multiple unpaired Student t-test for CD4^+^-depleted mice at week 2 (p < 0.01), week 3 (p < 0.05) and week 4 (p < 0.0001) and for CD8^+^-depleted mice at week 2 (p < 0.01), week 3 (p < 0.01) and week 4 (p < 0.000001) as compared to isotype group.

To provide further support to the role of T cells in protecting the mice from MOC2 tumor growth *in vivo*, we induced depletion of either CD4^+^ or CD8^+^ T cells by treating naïve mice with specific monoclonal antibodies, as described in Materials and Methods. As control, naïve mice were treated with irrelevant, isotype-matched monoclonal antibodies. Depletion of the relevant T cell subpopulation was measured by immunofluorescence and FACS analysis in spleen cells of sample mice (**Supplementary Figure S6**). It should be noted that while the depletion of CD8^+^ T lymphocytes was complete, treatment with the specific antibody resulted in an approximate 50% reduction of CD4^+^ T lymphocytes (**Supplementary Figure S6**). Treated mice were then injected with the same number of MOC2-CIITA tumor cells capable to induce protection and/or strong retardation in tumor growth, as described above (see **Figure 5**). Interestingly, while depletion with control antibody did not affect the protective immune response of the mice injected with MOC2-CIITA, depletion of CD4^+^ T cells and CD8^+^ T cells drastically reduced the immunogenic and protective effect against MOC2-CIITA tumors. Indeed, at four weeks post-tumor injection, mice depleted of CD4^+^ or CD8^+^ cells developed tumors, unlike MOC2-CIITA-injected mice, where 25% of the animals remained tumor-free (**Figure 8A**). The mean tumor size was 3.8-fold and 10-fold larger respectively as compared to that of control group (CD4^+^-depleted p<0.0001, CD8+-depleted p<0.000001) (**Figure 8B**). The less marked loss of protection observed after depletion of CD4^+^ T cells as compared to depletion of CD8^+^ T cells is most likely due to the residual presence of CD4^+^ T cells after the depletion treatment with the anti-CD4^+^ monoclonal antibody used for this experiment (**Supplementary Figure S6**).

## 4 Discussion

In recent years, innovative approaches in the field of cancer therapy have significatively changed the clinical scenario for patients affected by advanced cancer with poor prognosis. In the last decade the introduction of ICI in the treatment of non-small cell lung cancer (NSCLC) and melanoma has brought to substantially improved survival (42–44).

However, response to immunotherapy proved controversial in other types of cancer, as in the case of Head and Neck Squamous Cell Carcinoma (HNSCC), including Oral Squamous Cell Carcinoma (OSCC), the most common malignancy of the oral cavity. Indeed, clinical trials which analyzed the efficacy of ICI with anti-PD-1 therapy in recurrent and/or metastatic HNSCC showed that only 15–20% of patients benefit from this treatment, emphasizing the need for additional immunotherapeutic approaches, such as immunotherapeutic vaccines (45).

As CD8^+^ CTL are the major effectors of anti-tumor immunity, most of the studies of anti-tumor vaccines have focused on the selection of potential tumor antigen peptides that could bind to MHC-I molecules, as these cell surface molecules are the restricting elements for CTLs and are expressed in all cells, thus also in tumor cells. However, also this approach did not result in optimal clinical outcomes (18). For these reasons, several groups, including ours, have stressed the importance of focusing the attention on CD4^+^ TH, because without triggering of TH cells, effector CTLs cannot proliferate and be maintained for a long time, particularly *in vivo* (46).

Taken together, these considerations have guided us to investigate the possibility that an optimal stimulation of the tumor specific CD4^+^ TH cells could better activate the cascade of events leading to an efficient anti-tumor therapy in OSCC. Toward this goal, we decided first to better study the OSCC tumor microenvironment in terms of cell subpopulations and relevant markers of immunity to get hints of the importance of CD4^+^ TH cells in a clinical setting, and then to assess in an *in vivo* animal experimental model of OSCC the efficacy of an anti-tumor vaccination focused on the optimal activation of tumor specific TH cells.

In the tumor microenvironment, MHC-I antigens were constitutively expressed in all OSCC tumor cells, but with different intensity. The variable degree of MHC-I positivity reverberated on clinical outcomes, since stage IV oral cancers were significantly associated to the hypoexpression of this marker. This finding is consistent with those reported in literature and gains interest because in many cases tumor cells tend to lose expression of MHC-I molecules, and this has been correlated with mechanisms of escape from immune recognition by tumor specific CD8^+^ CTL (47). Moreover, the hypoexpression of MHC-I in tumor cells can also have a negative impact on adjuvant immunotherapy aimed at blocking immune checkpoint molecules in recurrent and/or metastatic OSCC (48).

On the other side, MHC-II molecules, the restricting element for CD4^+^ TH cell recognition, were detected in a relevant amount of tumor cells (at least 10%) only in 10 patients of the recruited population (i.e., 43.5%). The expression of MHC-II molecules in tumor cells is generally believed to be associated with a better prognosis (49). Even though this could not be confirmed in this case, the MHC-II-positive tumors displayed a significant CD4^+^ T lymphocyte infiltration, suggesting the involvement of an MHC-II-restricted CD4^+^ TH cell activation against tumor antigens.

Beside MHC expression, the OSCC tumor microenvironment was characterized by a high number of PD-1-positive immune cells infiltrating the tumor tissues and this was associated with a higher incidence of LN metastasis at diagnosis. PD-1-expressing cells were mainly CD8^+^ T lymphocytes in both tumor tissues and stroma. This indicates that OSCC tumors are indeed "hot tumors", a definition that has been recently proposed for tumor tissue that presents an inflamed status with relevant infiltration by leukocytes (50). This is an important element because indicates that an anti-tumor immune activation has been generated although functionally insufficient to counteract tumor growth.

While PD-1 is essentially expressed in functionally mature immune cells, its interacting ligand PD-L1 is usually expressed in APC as well as other cells, including tumor cells (51). In patients' tumor tissues, PD-L1 expression correlated with the total immune infiltrate in the stroma, but not with a specific cell phenotype, indicating the probable contribution by multiple cell subtypes. In our sample, about half of the tumors (i.e., 47.8%) were PD-L1-positive and, interestingly, PD-L1 positivity was found in the same cases expressing MHC-II molecules.

The co-expression of both MHC-II and PD-L1 molecules on OSCC tumor cells certainly represents a combination of two independent events, which however have in common the fact that they can be induced by IFN-γ (52, 53). In an inflamed tumor tissue, IFN-γ can be secreted by infiltrating lymphocytes and this can be at the basis of the concomitant expression of MHC-II and PD-L1 observed in our OSCC samples. Favoring the MHC-II restricted tumor antigen recognition by anti-tumor CD4^+^ TH cells from one side and blocking their functional activity by PD-1/PD-L1 interaction from the other side could partially explain the lack of clear beneficial outcomes in terms of survival in MHC-II-positive cancers (54). We may thus hypothesize that the PD-L1 checkpoint molecule could represent a viable target for immunotherapy especially in MHC-II-positive oral cancer, even though further experiments are required to confirm this association.

The findings described in our clinicopathological study were paralleled by similar results obtained in human OSCC cell lines CAL-27, SCC-25, and SCC-4. Indeed, all the three lines expressed MHC-I antigens on the cell surface, but MHC-II molecules were partially detected only in CAL-27, and not in SCC-25 and SCC-4. In addition, PD-L1 was expressed in CAL-27 and SCC-4, but not in SCC-25 cells. However, after treatment with IFN-γ, all the OSCC cell lines increased MHC-I expression and became MHC-II-positive and PD-L1-positive, confirming the role of the cytokine in rescuing the cell surface expression of these two markers (55).

Based on the above results and on the ground of our large preclinical studies on the effect of CIITA-mediated MHC-II expression in a variety of tumor histotypes of distinct MHC genetic background, we were strongly motivated to assess, in an experimental animal model, the OSCC murine cell line MOC2, whether a vaccination approach with CIITA-mediated, MHC-II-expressing OSCC tumor cells could similarly induce a protective immune response *in vivo* (56–58).

Moreover, previous results in a preclinical model of head and neck cancer by using a similar cell line, MOC22, as the one used in our study, revealed tumor growth retardation in mice injected with a synthetic long peptide (SLP) with affinity for both MHC-I and MHC-II epitopes (mICAM1) with respect to results obtained with a SLP with affinity limited to only MHC-I antigens (p15E) (59).

We generated MOC2-CIITA stable transfectants expressing MHC-II molecules; of note, these transfected cells did not express PD-L1 on their surface. MOC2-CIITA cells were then injected into syngeneic immunocompetent C57BL/6 mice. We clearly showed that the modified MOC2-CIITA tumor cells were strongly immunogenic *in vivo* as compared to highly tumorigenic parental MOC2 cells. 40% of MOC2-CIITA injected mice rejected the tumor, while the remaining 60% displayed a strongly retarded tumor growth. Interestingly, *ex vivo* analysis of MOC2-CIITA tumor growing *in vivo* showed a clear reduction of MHC-II expression, thus strongly correlating anti-tumor capacity to respond *in vivo* to the amount of CIITA-dependent MHC-II expression. It must be underlined that relative reduction of MHC-II expression *in vivo* has been observed also in previous studies by our group. This event is possibly due to two reasons: first, downregulation of the expression of the CIITA transfected plasmid (24) whose molecular mechanisms are unclear at present, or alternatively to the growth selection *in vivo* of a CIITA-transfected population with a low expression of CIITA and by consequence a low expression of MHC-II molecules. Continuous cloning of the CIITA-transfected cells and selection of more stable and highly MHC-II-expressing transfectants can overcome the problem and result in a more efficient rejection *in vivo* of the modified tumor cells, as we have shown in previous analyses (57).

Phenotypic analysis of spleen cells from animals protected after vaccination with MOC2-CIITA tumor cells showed that this event was associated to a preferential skewing toward a TH1 type of immune response, with respect to a polarization toward a TH2-like phenotype observed in animals with MOC2 parental growing tumors. These results are in line with our previously published results in other tumor models (57), suggesting a preferential triggering of TH1 responses following vaccination with CIITA-modified tumor cells.

The immunogenic nature of the rejection of MOC2-CIITA cells was demonstrated by the acquisition of a protective memory response to subsequent challenge with parental MOC2 cell line.

To further characterize the specific immune response elicited by our vaccination protocol, we performed an adoptive cell transfer (ACT) experiment by harvesting total immune cells, CD4^+^ or CD8^+^ T lymphocytes from the spleen of vaccinated mice that have rejected tumors and selectively co-injected these cells with unmodified MOC2 cells into naïve mice. The experiment demonstrated that both CD4^+^ and CD8^+^ T lymphocytes confer resistance to naïve animals and induce tumor rejection/growth retardation, with the CD4^+^ T cells representing the most effective cell subtype. In addition, we further verified the role played by both CD4^+^ and CD8^+^ T cells in anti-tumor response elicited after the vaccination protocol by performing an *in vivo* depletion experiment, e.g., depleting selectively CD4^+^ or CD8^+^ T cells while injecting MOC2-CIITA or parental cells in mice. The findings described in this study highlighted that the depletion of CD4^+^ or CD8^+^ T cells in MOC2-CIITA vaccinated mice resulted in the nullification of the immune response against tumors, as MOC2-CIITA tumor cells were growing with a similar kinetics as the MOC2 parental cells.

Taken together, our MOC2-CIITA vaccination results demonstrate that for OSCC tumors the CIITA-mediated MHC-II expression is instrumental to trigger the initial phase of the adaptive immune response, particularly the key triggering of tumor specific CD4^+^ T cells that in turn activate and allow the functional maturation of CD8^+^ tumor-specific CTL. They also emphasize once more the surrogate antigen presentation activity of CIITA-dependent MHC-II expression in tumor cells, as it has been previously demonstrated by our group in other *in vivo* tumor models (25).

We believe that our studies are of relevance for future strategies of immune intervention in OSCC clinical setting. The success of immune vaccination with CIITA-modified OSCC tumor cells opens the possibility to use these cells to isolate MHC-II-bound tumor antigenic peptides that can serve for the preparation of novel therapeutic vaccines against OSCC tumors (60). Moreover, approaches to modify *in vivo* OSCC tumors by injecting CIITA into the tumor tissues may help to increase the immunogenicity of tumors otherwise scarcely immunogenic. In this regard, viral vectors containing CIITA can be optimized to vehicle the MHC class II transactivator directly and specifically into OSCC tumor mass (61), as this tumor is easily accessible for direct injection.

In conclusion, CIITA modification of tumor cells alone or in synergy with immune checkpoint blockade could soon represent a suitable alternative for the treatment of oral cancer.

## Data availability statement

The raw data supporting the conclusions of this article will be made available by the authors, without undue reservation.

## Ethics statement

The studies involving humans were approved by local Hospital Ethical Committee (Comitato Etico dell'Insubria). The studies were conducted in accordance with the local legislation and institutional requirements. Written informed consent for participation was not required from the participants or the participants' legal guardians/next of kin because retrospective histopathological study. The animal study was approved by local Animal Welfare Ethical Committee (OPBA), Italian Ministry of Health, protocol n° 249/2022-PR. The study was conducted in accordance with the local legislation and institutional requirements.

## Author contributions

LA: Data curation, Formal analysis, Investigation, Methodology, Writing – original draft, Visualization. FC: Data curation, Investigation, Software, Writing – review & editing. AC: Methodology, Supervision, Writing – review & editing. AS: Formal analysis, Investigation, Software, Writing – review & editing. MS: Formal analysis, Investigation, Software, Writing – review & editing. AG: Data curation, Writing – review & editing. PB: Data curation, Supervision, Writing – review & editing. SL: Methodology, Resources, Writing – review & editing. AT: Funding acquisition, Project administration, Resources, Writing – review & editing. RA: Conceptualization, Project administration, Supervision, Validation, Writing – review & editing. GF: Conceptualization, Investigation, Methodology, Project administration, Resources, Software, Validation, Visualization, Writing – original draft, Writing – review & editing.

## References

[1] Chamoli A Gosavi AS Shirwadkar UP Wangdale KV Behera SK Kurrey NK. Overview of oral cavity squamous cell carcinoma: risk factors, mechanisms, and diagnostics. Oral Oncol. (2021) 121:105451. doi: 10.1016/j.oraloncology.2021.105451 34329869

[2] Global Cancer Observatory. Available online at: https://gco.iarc.fr/ (Accessed October 30, 2023).

[3] Wyss A Hashibe M Chuang SC Lee YC Zhang ZF Yu GP. Cigarette, cigar, and pipe smoking and the risk of head and neck cancers: pooled analysis in the International Head and Neck Cancer Epidemiology Consortium. Am J Epidemiol. (2013) 178:679–90. doi: 10.1093/aje/kwt029 PMC3755640 23817919

[4] Bagnardi V Rota M Botteri E Tramacere I Islami F Fedirko V. Alcohol consumption and site-specific cancer risk: a comprehensive dose-response meta-analysis. Br J Cancer. (2015) 112:580–93. doi: 10.1038/bjc.2014.579 PMC4453639 25422909

[5] Wyss AB Hashibe M Lee YA Chuang SC Muscat J Chen C. Smokeless tobacco use and the risk of head and neck cancer: pooled analysis of US studies in the INHANCE consortium. Am J Epidemiol. (2016) 184:703–16. doi: 10.1093/aje/kww075 PMC5141945 27744388

[6] Dahlstrom KR Little JA Zafereo ME Lung M Wei Q Sturgis EM. Squamous cell carcinoma of the head and neck in never smoker-never drinkers: a descriptive epidemiologic study. Head Neck. (2008) 30:75–84. doi: 10.1002/hed.20664 17694557

[7] Tran Q Maddineni S Arnaud EH Divi V Megwalu UC Topf MC. Oral cavity cancer in young, non-smoking, and non-drinking patients: a contemporary review. Crit Rev Oncol Hematol. (2023) 190:104112. doi: 10.1016/j.critrevonc.2023.104112 37633348 PMC10530437

[8] Szturz P Vermorken JB. Management of recurrent and metastatic oral cavity cancer: raising the bar a step higher. Oral Oncol. (2020) 101:104492. doi: 10.1016/j.oraloncology.2019.104492 31837576

[9] Zhang X Wang P Chai Y Zhou X Li P Wang X. Real-world data of immunotherapy from China in recurrent or metastatic head and neck squamous cell carcinoma. Am J Otolaryngol. (2023) 45:104065. doi: 10.1016/j.amjoto.2023.104065 37879241

[10] Grossi F Crinò L Logroscino A Canova S Delmonte A Melotti B. Use of nivolumab in elderly patients with advanced squamous non-small cell lung cancer: results from the Italian cohort of an expanded access programme. Eur J Cancer. (2018) 100:126–34. doi: 10.1016/j.ejca.2018.05.015 30014881

[11] Cramer JD Burtness B Ferris RL. Immunotherapy for head and neck cancer: recent advances and future directions. Oral Oncol. (2019) 99:104460. doi: 10.1016/j.oraloncology.2019.104460 31683169 PMC7749717

[12] Nindra U Hurwitz J Forstner D Chin V Gallagher R Liu J. A systematic review of neoadjuvant and definitive immunotherapy in locally advanced head and neck squamous cell carcinoma. Cancer Med. (2023) 12:11234–47. doi: 10.1002/cam4.5815 PMC10242857 36934434

[13] Bou Nasser Eddine F Ramia E Tosi G Forlani G Accolla RS. Tumor immunology meets … immunology: modified cancer cells as professional APC for priming naïve tumor-specific CD4+ T cells. Oncoimmunology. (2018) 8:1548243. doi: 10.1080/2162402X.2017.1356149 29147609 PMC5674956

[14] Gulley JL Madan RA Tsang KY Jochems C Marté JL Farsaci B. Immune impact induced by PROSTVAC (PSA-TRICOM), a therapeutic vaccine for prostate cancer. Cancer Immunol Res. (2014) 2:133–41. doi: 10.1158/2326-6066.CIR-13-0108 PMC4004961 24778277

[15] Garcia-Lora A Algarra I Garrido F. MHC class I antigens, immune surveillance, and tumor immune escape. J Cell Physiol. (2003) 195:346–55. doi: 10.1002/jcp.10290 12704644

[16] DhatChinamoorthy K Colbert JD Rock KL. Cancer immune evasion through loss of MHC class I antigen presentation. Front Immunol. (2021) 12:636568. doi: 10.3389/fimmu.2021.636568 33767702 PMC7986854

[17] Koike K Dehari H Shimizu S Nishiyama K Sonoda T Ogi K. Prognostic value of HLA class I expression in patients with oral squamous cell carcinoma. Cancer Sci. (2020) 111:1491–9. doi: 10.1111/cas.14388 PMC7226222 32167621

[18] Buonaguro L Tagliamonte M. Peptide-based vaccine for cancer therapies. Front Immunol. (2023) 14:1210044. doi: 10.3389/fimmu.2023.1210044 37654484 PMC10467431

[19] Holling TM Schooten E van Den Elsen PJ. Function and regulation of MHC class II molecules in T-lymphocytes: of mice and men. Hum Immunol. (2004) 65:282–90. doi: 10.1016/j.humimm.2004.01.005 15120183

[20] Giacomini P Fisher PB Duigou GJ Gambari R Natali PG. Regulation of class II MHC gene expression by interferons: insight into the mechanism of action of interferon (review). Anticancer Res. (1988) 8:1153–61. 2464333

[21] Axelrod ML Cook RS Johnson DB Balko JM. Biological consequences of MHC-II expression by tumor cells in cancer. Clin Cancer Res. (2019) 25:2392–402. doi: 10.1158/1078-0432.CCR-18-3200 PMC6467754 30463850

[22] Accolla RS Jotterand-Bellomo M Scarpellino L Maffei A Carra G Guardiola J. Air-1, a newly found locus on mouse chromosome 16 encoding a trans-acting activator factor for MHC class II gene expression. J Exp Med. (1986) 164:369–74. doi: 10.1084/jem.164.1.369 PMC2188193 3088202

[23] Accolla RS Lombardo L Abdallah R Raval G Forlani G Tosi G. Boosting the MHC class II-restricted tumor antigen presentation to CD4+ T helper cells: a critical issue for triggering protective immunity and re-orienting the tumor microenvironment toward an anti-tumor state. Front Oncol. (2014) 4:32. doi: 10.3389/fonc.2014.00032 24600588 PMC3927100

[24] Meazza R Comes A Orengo AM Ferrini S Accolla RS. Tumor rejection by gene transfer of the MHC class II transactivator in murine mammary adenocarcinoma cells. Eur J Immunol. (2003) 33:1183–92. doi: 10.1002/eji.200323712 12731043

[25] Bou Nasser Eddine F Forlani G Lombardo L Tedeschi A Tosi G Accolla RS. CIITA-driven MHC class II expressing tumor cells can efficiently prime naïve CD4^+^ TH cells *in vivo* and vaccinate the host against parental MHC-II-negative tumor cells. Oncoimmunology. (2017) 6:e1261777. doi: 10.1080/2162402X.2016.1261777 28197387 PMC5283634

[26] Ridge JA Lydiatt WM Patel SG Glastonbury CM Brandwein-Gensler M Ghossein RA. “Oral cavity”. In: AJCC Cancer Staging Manual, Eight Edition. New York: Springer (2017). p. 79–94. doi: 10.1007/978-3-319-40618-3_7

[27] WHO Classification of Tumours Editorial Board. Head and neck tumours. 5th ed Vol. 9. . Lyon (France: International Agency for Research on Cancer (2022). WHO classification of tumours series.

[28] Kakkar A Thakur R Roy D Sood R Sharma A Malhotra RK. Tumour-infiltrating lymphocyte subsets and their individual prognostic impact in oral squamous cell carcinoma. J Clin Pathol. (2023) jcp–2023–208918. doi: 10.1136/jcp-2023-208918 37699696

[29] Paver EC Cooper WA Colebatch AJ Ferguson PM Hill SK Lum T. Programmed death ligand-1 (PD-L1) as a predictive marker for immunotherapy in solid tumours: a guide to immunohistochemistry implementation and interpretation. Pathology. (2021) 53:141–56. doi: 10.1016/j.pathol.2020.10.007 33388161

[30] Chiaravalli AM Longhi E Vigetti D De Stefano FI Deleonibus S Capella C. Gastrointestinal cancers reactive for the PAb416 antibody against JCV/SV40 T-Ag lack JCV DNA sequences while showing a distinctive pathological profile. J Clin Pathol. (2012) 66:44. doi: 10.1136/jclinpath-2012-200963 23012397

[31] Gioanni J Fischel JL Lambert JC Demard F Mazeau C Zanghellini E. Two new human tumor cell lines derived from squamous cell carcinomas of the tongue: establishment, characterization and response to cytotoxic treatment. Eur J Cancer Clin Oncol. (1988) 24:1445–55. doi: 10.1016/0277-5379(88)90335-5 3181269

[32] Rheinwald JG Beckett MA. Tumorigenic keratinocyte lines requiring anchorage and fibroblast support cultures from human squamous cell carcinomas. Cancer Res. (1981) 41:1657–63. 7214336

[33] Kono M Saito S Egloff AM Allen CT Uppaluri R. The mouse oral carcinoma (MOC) model: a 10-year retrospective on model development and head and neck cancer investigations. Oral Oncol. (2022) 132:106012. doi: 10.1016/j.oraloncology.2022.106012 35820346 PMC9364442

[34] Judd NP Winkler AE Murillo-Sauca O Brotman JJ Law JH Lewis JS Jr. ERK1/2 regulation of CD44 modulates oral cancer aggressiveness. Cancer Res. (2012) 72:365–74. doi: 10.1158/0008-5472.CAN-11-1831 PMC3286642 22086849

[35] Lemonnier FA Rebai N Le Bouteiller PP Malissen B Caillol DH Kourilsky FM. Epitopic analysis of detergent-solubilized HLA molecules by solid-phase radioimmunoassay. J Immunol Methods. (1982) 54:9–22. doi: 10.1016/0022-1759(82)90108-9 6183366

[36] Carrel S Tosi R Gross N Tanigaki N Carmagnola AL Accolla RS. Subsets of human Ia-like molecules defined by monoclonal antibodies. Mol Immunol. (1981) 18:403–11. doi: 10.1016/0161-5890(81)90102-4 6171715

[37] Scupoli MT Sartoris S Tosi G Ennas MG Nicolis M Cestari T. Expression of MHC class I and class II antigens in pancreatic adenocarcinomas. Tissue Antigens. (1996) 48:301–11. doi: 10.1111/j.1399-0039.1996.tb02649.x 8946684

[38] Ramia E Chiaravalli AM Bou Nasser Eddine F Tedeschi A Sessa F Accolla RS. and heterogeneous expression of immune checkpoints in hepatocarcinomas: implications for new therapeutic approaches. Oncoimmunology. (2018) 8:1548243. doi: 10.1080/2162402X.2018.1548243 30723578 PMC6350839

[39] Dutta T Spence A Lampson LA. Robust ability of IFN-gamma to upregulate class II MHC antigen expression in tumor bearing rat brains. J Neurooncol. (2003) 64:31–44. doi: 10.1007/BF02700018 12952284

[40] Tosi G Forlani G Andresen V Turci M Bertazzoni U Franchini G. Major histocompatibility complex class II transactivator CIITA is a viral restriction factor that targets human T-cell lymphotropic virus type 1 Tax-1 function and inhibits viral replication. J Virol. (2011) 85:10719–29. doi: 10.1128/JVI.00813-11 PMC3187506 21813598

[41] Celesti F Gatta A Shallak M Chiaravalli AM Cerati M Sessa F. Protective anti-tumor vaccination against glioblastoma expressing the MHC class II transactivator CIITA. Front Immunol. (2023) 14:1133177. doi: 10.3389/fimmu.2023.1133177 36993983 PMC10040613

[42] Pasqualotto E de Moraes FCA Pedrotti Chavez M Souza MEC Rodrigues ALSO Ferreira ROM. PD-1/PD-L1 inhibitors plus chemotherapy versus chemotherapy alone for resectable non-small cell lung cancer: a systematic review and meta-analysis of randomized controlled trials. Cancers (Basel). (2023) 15:5143. doi: 10.3390/cancers15215143 37958317 PMC10648147

[43] Mahdiabadi S Momtazmanesh S Katimi A Rezaei N. Immune checkpoint inhibitors in advanced cutaneous melanoma: a systematic review and meta-analysis of efficacy and review of characteristics. Expert Rev Anticancer Ther. (2023) 23:1281–93. doi: 10.1080/14737140.2023.2278509 37908134

[44] Fitzsimmons TS Singh N Walker TDJ Newton C Evans DGR Crosbie EJ. Immune checkpoint inhibitors efficacy across solid cancers and the utility of PD-L1 as a biomarker of response: a systematic review and meta-analysis. Front Med (Lausanne). (2023) 10:1192762. doi: 10.3389/fmed.2023.1192762 37250628 PMC10219231

[45] Harrington KJ Ferris RL Gillison M Tahara M Argiris A Fayette J. Efficacy and safety of nivolumab plus ipilimumab vs nivolumab alone for treatment of recurrent or metastatic squamous cell carcinoma of the head and neck: the Phase 2 CheckMate 714 randomized clinical trial. JAMA Oncol. (2023) 9:779–89. doi: 10.1001/jamaoncol.2023.0147 PMC10080406 37022706

[46] Borst J Ahrends T Bąbała N Melief CJM Kastenmüller W. CD4 (+) T cell help in cancer immunology and immunotherapy. Nat Rev Immunol. (2018) 18:635–47. doi: 10.1038/s41577-018-0044-0 30057419

[47] Wickenhauser C Bethmann D Kappler M Eckert AW Steven A Bukur J. Tumor microenvironment, HLA class I and APM expression in HPV-negative oral squamous cell carcinoma. Cancers (Basel). (2021) 13:620. doi: 10.3390/cancers13040620 33557271 PMC7914856

[48] Jiang N Yu Y Wu D Wang S Fang Y Miao H. HLA and tumour immunology: immune escape, immunotherapy and immune-related adverse events. J Cancer Res Clin Oncol. (2023) 149:737–47. doi: 10.1007/s00432-022-04493-1 PMC11797339 36662304

[49] Samuels S Spaans VM Osse M Peters LAW Kenter GG Fleuren GJ. Human Leukocyte Antigen-DR expression is significantly related to an increased disease-free and disease-specific survival in patients with cervical adenocarcinoma. Int J Gynecol Cancer. (2016) 26:1503–9. doi: 10.1097/IGC.0000000000000783 27654088

[50] Chen DS Mellman I. Elements of cancer immunity and the cancer-immune set point. Nature. (2017) 541:321–30. doi: 10.1038/nature21349 28102259

[51] Zheng Y Fang YC Li J. PD-L1 expression levels on tumor cells affect their immunosuppressive activity. Oncol Lett. (2019) 18:5399–407. doi: 10.3892/ol.2019.10903 PMC6781757 31612048

[52] Baleeiro RB Bouwens CJ Liu P Di Gioia C Dunmall LSC Nagano A. MHC class II molecules on pancreatic cancer cells indicate a potential for neo-antigen-based immunotherapy. Oncoimmunol. (2022) 11:2080329. doi: 10.1080/2162402X.2022.2080329 PMC9154752 35655709

[53] Zibelman M MacFarlane AW4 Costello K McGowan T O’Neill J Kokate R. A phase 1 study of nivolumab in combination with interferon-gamma for patients with advanced solid tumors. Nat Commun. (2023) 14:4513. doi: 10.1038/s41467-023-40028-z 37500647 PMC10374608

[54] Costantini F Barbieri G. The HLA-DR mediated signalling increases the migration and invasion of melanoma cells, the expression and lipid raft recruitment of adhesion receptors, PD-L1 and signal transduction proteins. Cell Signal. (2017) 36:189–203. doi: 10.1016/j.cellsig.2017.05.008 28495591

[55] Mutlu S Sculy C Prime SS. Effect of IFN-gamma on the expression of MHC class I and class II antigens in a human Malignant oral epithelial cell line. J Oral Pathol Med. (1991) 20:218–21. doi: 10.1111/j.1600-0714.1991.tb00422.x 1906105

[56] Accolla RS Ramia E Tedeschi A Forlani G. CIITA-driven MHC class II expressing tumor cells as antigen presenting cell performers: toward the construction of an optimal anti-tumor vaccine. Front Immunol. (2019) 10:1806. doi: 10.3389/fimmu.2019.01806 31417570 PMC6682709

[57] Mortara L Castellani P Meazza R Tosi G De Lerma Barbaro A Procopio FA. CIITA-induced MHC class II expression in mammary adenocarcinoma leads to a Th1 polarization of the tumor microenvironment, tumor rejection, and specific antitumor memory. Clin Cancer Res. (2006) 12:3435–43. doi: 10.1158/1078-0432.CCR-06-0165 16740768

[58] Forlani G Shallak M Celesti F Accolla RS. Unveiling the hidden treasury: CIITA-driven MHC class II expression in tumor cells to dig up the relevant repertoire of tumor antigens for optimal stimulation of tumor specific CD4+ T helper cells. Cancers (Basel). (2020) 12:3181. doi: 10.3390/cancers12113181 33138029 PMC7693840

[59] Shibata H Xu N Saito S Zhou L Ozgenc I Webb J. Integrating CD4+ T cell help for therapeutic cancer vaccination in a preclinical head and neck cancer model. Oncoimmunology. (2021) 10:1958589. doi: 10.1080/2162402X.2021.1958589 34408919 PMC8366550

[60] Forlani G Michaux J Pak H Huber F Marie Joseph EL Ramia E. CIITA-transduced glioblastoma cells uncover a rich repertoire of clinically relevant tumor-associated HLA-II antigens. Mol Cell Proteomics. (2021) 20:100032. doi: 10.1074/mcp.RA120.002201 33592498 PMC8724627

[61] Martikainen M Essand M. Virus-based immunotherapy of glioblastoma. Cancers (Basel). (2019) 11:186. doi: 10.3390/cancers11020186 30764570 PMC6407011

