# Supplementary material for: Novel vaccination strategies based on optimal stimulation of CD4+ T helper cells for the treatment of oral squamous cell carcinoma
Source: Front Immunol. 2024 Jul 5;15:1387835. doi: 10.3389/fimmu.2024.1387835 (PMC11257872; doi:10.3389/fimmu.2024.1387835)
Supplement: Supplementary file 1 [file DataSheet_1.pdf]

Supplementary Appendix

Novel vaccination strategies based on optimal stimulation of $CD4^+$ T helper cells for the treatment of Oral Squamous Cell Carcinoma.

MATERIALS AND METHODS……………………………………………………………………2

Table S1. Conditions and reagents used for immunohistochemical study…….……………………………………………2

Table S2. Scoring parameters applied for immunohistochemical study…………………………………………………2

Table S3. Cell lines and related reagents used in this study………………………………………………………………3

Table S4. Antibodies and reagents used for FACS analysis………………………………………………. ….…..3

Quantification of mRNA by real-time PCR (qRT-PCR) and RT-PCR……………………………………………..4

In vitro assessment of T cell phenotype………………………………………………………………………….....4

RESULTS……………………………………………………………………………………5

Figure S1. CIITA mRNA transcription is induced after IFN-γ treatment in human OSCC cell lines……………..……..5

Figure S2. IFN-γ treatment induces CIITA gene trascription in MOC2 cells ..……………………………………......6

Figure S3. MOC2-CIITA kinetics of growth is not altered after CIITA transfection ………………….......................6

Figure S4. The stable expression of pAIP empty vector in MOC2 cells did not affect tumor growth *in vivo*. …………..7

Figure S5. In vitro assessment of T cell phenotype ..……………………………………………………………………8

Figure S6. Efficiency of both CD4 and CD8 T cells in vivo depletion ……………...……………………………………9

MATERIALS AND METHODS

Table S1. Conditions and reagents used for immunohistochemical study.

**Antibody specificity**	**Source**	**M/R (Clone)**[a]	**Antigen retrieval**[b]	**Working dilution**
MHC-I	AbCam	R (EP1395Y)	TC (10 min)	1/250
MHC-II (DR)	ThermoFisher	M (LN-3)	TC (10 min)	1/300
$CD4^+$	Ventana	R (SP35)	E (10 min)	1/1
$CD8^+$	Ventana	R (SP57)	TC (10 min)	1/1
$CD68^+$	DAKO	M (1G12)	TC (10 min)	1/100
PD-1	Origene	M (UMAB199)	E (20 min)	1/500
PD-L1	Ventana	R (SP142)	E (20 min)	undiluted

a: M, mouse; R, rabbit.
b: TC, citrate buffer pH 6.0; E, EDTA pH 8.0; in parenthesis, microwave unmasking time.

Table S2. Scoring parameters applied for immunohistochemical study.

Antigen	***T/S***[a]	***TC/IC***[b]	***Scoring parameters***
MHC-I	T	TC	low expression (< 50%); moderate expression (50 ≤ % < 90); high expression (≥ 90%)
MHC-II	T	TC	absent (< 10%); expressed (≥ 10%)
$CD4^+$	T	IC	low expression (< 5 cells/HPF); high expression (≥ 5 cells/HPF)
	S	IC	low expression (< 10 cells/HPF); high expression (≥ 10 cells/HPF)
$CD8^+$	T	IC	low expression (< 5 cells/HPF); high expression (≥ 5 cells/HPF)
	S	IC	low expression (< 10 cells/HPF); high expression (≥ 10 cells/HPF)
$CD68^+$	T	IC	low expression (< 5 cells/HPF); high expression (≥ 5 cells/HPF)
	S	IC	low expression (< 10 cells/HPF); high expression (≥ 10 cells/HPF)
PD-1	T	IC	low expression (< 10 cells/HPF); high expression (≥ 10 cells/HPF)
	S	IC	low expression (< 20 cells/HPF); high expression (≥ 20 cells/HPF)
PD-L1[c,d]	T	TC	absent (TPS[c] < 10%); expressed (TPS ≥ 10%)
	T/S	IC	low expression (CPS[d] < 10); high expression (CPS ≥ 10)

a: Tumor tissue (T) or Stroma (S).
b: Tumor cells (TC) or Immune cells (IC).
c: Tumor Proportion Score (TPS): percentage of PD-L1-positive tumor cells on total tumor cells
d: Combined Positive Score (CPS): [Number of PD-L1-positive (tumor, lymphocyte, and macrophage) cells / Total number of viable cells)]*100.

Table S3. Cell lines and related reagents used in this study.

	Source	*Cat. No*
Cell line		
CAL-27	ATCC	CRL-2095
SCC-25	ATCC	CRL-1628
SCC-4	ATCC	CRL-1624
MOC2	Kerafast Inc.	EWL002-FP
Reagents		
DMEM	ATCC	30-2002
DMEM:F12	ATCC	30-2006
MITC-STO (ATCC 56-X)	ATCC	56-X.2™
IMDM	HyClone	SH30228.02
Ham's F12	HyClone	SH30026.01
FBS	Merck	F7524
Penn-Strep	Fisher Scientific	BW17-603E
Filter Flask	Fisher Scientific	09-761-108
Insulin	Sigma Aldrich	I6634-50mg
Hydrocortisone	Sigma Aldrich	H0135-1mg
EGF	Sigma Aldrich	01-107

Table S4. Antibodies and reagents used for FACS analysis.

	Source	*Cat. No*
Primary Antibody		
Human OSCC cell lines (CAL-27, SCC-25, SCC-4)		
B9.12.1 (anti-MHC-I), FITC	-	SN[a]
D1-12 (anti-MHC-II-DR), FITC	-	SN[a]
Anti-human CD279 (PD-1), EH12.2H7, PE	BioLegend	329906
Anti-human CD274 (B7-H1, PD-L1), 29E.2A3, PE	BioLegend	329706
Murine MOC2 OSCC cell line		
M1/42 (anti-mouse H-2), FITC	BioLegend	125508
M5/114.15.2 (anti-mouse I-A/I-E), PerCP/Cyanine5.5	BioLegend	107656
CD279 (anti-mouse PD-1), J43, PE	eBioscience	12-9985-82
CD274 (anti-mouse PD-L1, B7-H1), MIH5, PE	eBioscience	12-5982-82
Reagent		
Interferon gamma (IFNG), Human recombinant protein	OriGene	TP723709

a. SN = supernatant.

Quantification of mRNA by real-time PCR (qRT-PCR) and RT-PCR

Total RNA, was extracted from cells using TRIzol reagent (Thermo Fisher Scientific, catalog number 15596026), as previously described (Ramia E, Chiaravalli AM, Bou Nasser Eddine F, et al, ref 38 maintext). cDNA was synthesized from 0.5 μg total RNA using iScript cDNA Synthesis Kit (Bio-Rad, catalog number 170–8890). 0.5 μg of cDNA were amplified by PCR by using an ABI Prism 7000 sequence detection system (Thermo Fisher Scientific) with IQSYBR Green PCR master mix (Bio-Rad, catalog number 172–5122) according to the manufacturer protocol. All the reactions were performed in an ABI PRISM 7700 apparatus (PE Applied Biosystems). The PCR conditions were 50°C for 2 min, 95°C for 15 min, followed by 40 cycles at 95°C for 15 s and 60°C for 1 min. A 20 min dissociation protocol was also applied. The copy numbers of the CIITA, USF1 and IRF1 transcripts were cal- culated using the comparative Ct method (also known as the $2^{-\text{[delta] [delta]Ct}}$ method), where [delta]Ct, sample = Ct, CIITA/ USF1/IRF1 – Ct, RPS7, and [delta]Ct, sample is the Ct value for any sample normalised to the RPS7 endogenous house- keeping transcripts and [delta] [delta]Ct = [delta]Ct, sample – [delta]Ct, wt. In all the samples, $2^{-\text{[delta] [delta]Ct}}$ refers to an N-fold increase in the CIITA/IRF1 copy numbers relative to the untreated cells (vehicle). Each reaction was performed in triplicate. The following primer pair sets were used: CIITA forward 5′-ggatcctcacggcctttt-3′; reverse 5′- ccccgatcttgttctcactc-3. For RT-PCR analysis, 24 hours post IFNγ treatment, total RNA was extracted from MOC2 cells, reverse-transcribed and PCR-amplified with the following primers (5- cacccccagatgtgtatgtgct-3’, 5’acgaggtttcccagtccagaa-3’). The RT-PCR products were separated by agarose gel and visualized by Gel Red reagent (Biotium).

In vitro assessment of T cell phenotype

Spleen cells from animals showing tumor growth after injection of MOC-2 parental cells (Spleen tumor) or resulting protected from tumor after vaccination with MOC-2-CIITA (Spleen protected) were analyzed by ELISA assay to assess their TH2-like (IL-4 secretion) or TH1-like (IFNγ secretion) phenotype. Briefly, the read-out of TH1 or TH2 immune response was performed through co-culture of splenocytes coming from MOC2-pc bearing mouse and MOC2-CIITA vaccinated mouse at week 4 with either MOC2-pc or MOC2-CIITA tumor cells at a ratio of (1:5; effector:stimulator cells) in 96 well round-bottomed plate. Seventy-two hours later, supernatants of the co-culture were collected for cytokine secretion ELISA assays and compared to the supernatants of the seeded splenocytes or tumor cells alone. The cytokines production in the collected supernatants of splenocytes were measured using sandwich in-direct ELISA Flex: mouse IFN-γ (HRP) and mouse IL-4 (HRP) kits (Mabtech) according to the manufacturer’s instructions. The optical density was measured using AMR-100

microplate reader at 450nm. The cytokines concentration was then calculated based on the standard curve of each corresponding cytokine.

RESULTS

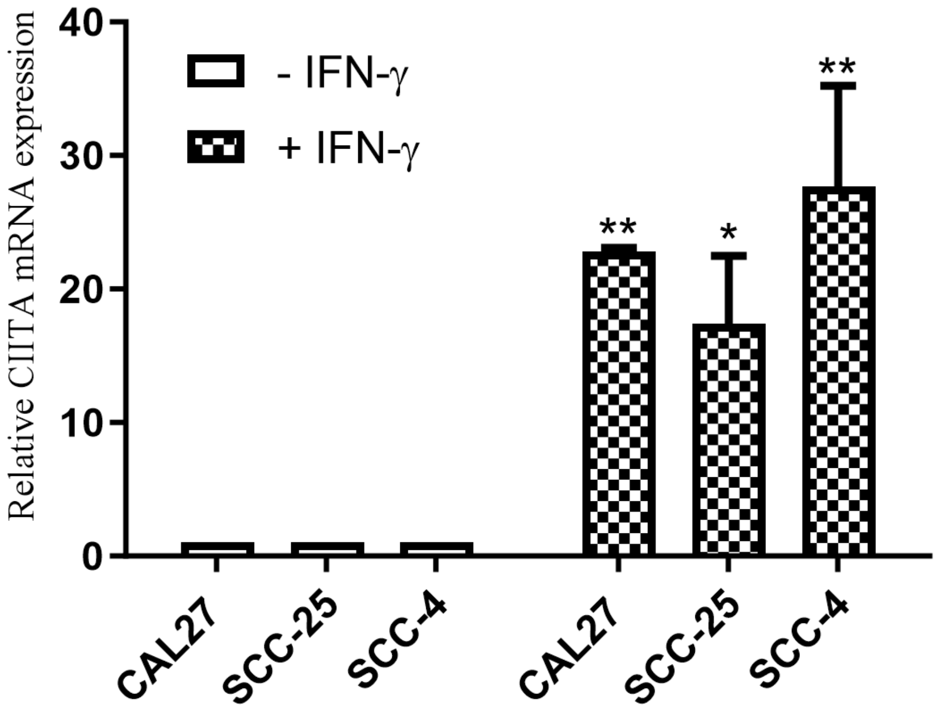

Figure S1. *CIITA mRNA transcription is induced after IFN-γ treatment in human OSCC cell lines.* CIITA mRNA expression in CAL-27, SCC-25, and SCC-4 OSCC cell lines 72 hours after treatment with IFN-γ (+, *checkered columns*) or its vehicle (-, *white columns*) assessed by qRT-PCR. The results of three representative experiments performed in triplicates are shown. CIITA mRNA levels in IFN-γ-treated cells are expressed as values relative to those of untreated cells to 1. Paired t-test has been performed (** $p \leq 0.01$ for CAL-27 and SCC-4, * $p \leq 0.05$ for SCC-25, as compared to corresponding untreated cells). Error bars represent the standard deviation.

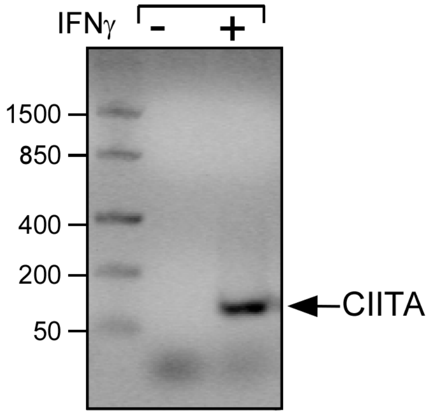

Figure S2. *IFN-γ treatment induces CIITA gene transcription in MOC2 cells.* 2x10^5 MOC2 parental cells were plated in 6 multi-well plates, the following day cells were treated with 1,000 U/mL of IFN-γ. Twenty-four hours post treatment, total RNA was extracted from the cells using TRIZOL reagent following the manufacturer's instructions. cDNA was synthesized from 1μg of total RNA using iScript™ cDNA Synthesis Kit (BIO-RAD). The amplicon representing CIITA transcripts is indicated by filled arrow.

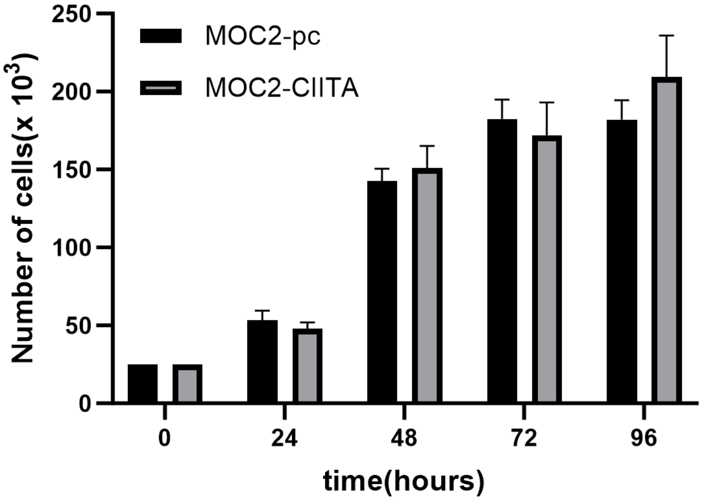

Figure S3. *MOC2-CIITA kinetics of growth is not altered after CIITA transfection.*

The histograms show the growth rate of both MOC2 parental cell line (MOC2pc) and CIITA-transfected, MHC-II-expressing MOC2 cell line (MOC2-CIITA). As the results clearly showed, the kinetics of growth of the two cell lines was comparable after the transfection of CIITA, and differences were not statistically significative after 96 hours (p=0.18). p-value was calculated *via* unpaired Student t-test. Number of cells counted (ordinate) over time (abscissa).

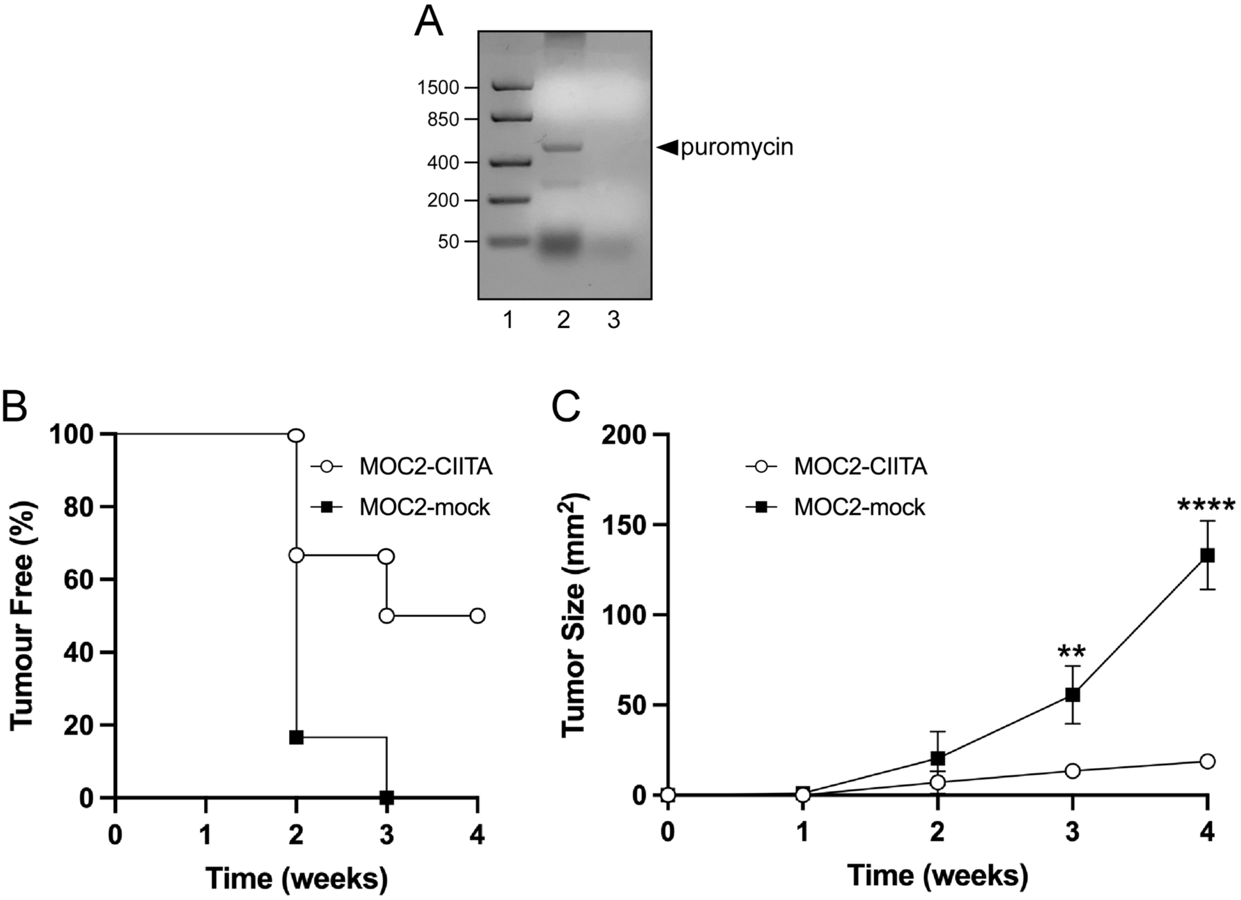

Figure S4. *The stable expression of pAIP empty vector in MOC2 cells did not affect tumor growth in vivo.* MOC2-CIITA tumors were rejected or strongly retarded in their growth after s.c injection in C57BL/6 mice (see Materials and Methods). (**A**) The expression of pAIP empty vector was assessed by RT-PCR for the presence of puromycin cassette. A band of about 500 bp was detected in MOC2-mock cells (Lane 2) but not in MOC2 parental cells (lane 3). The amplicon representing puromycin transcripts is indicated by the arrowhead. Lane 1 corresponds to DNA ladder (FastRuler Low Range DNA Ladder Thermo-scientific, Catalog number: SM1103) (**B**) The Kaplan-Meyer curve shows that 50% of mice that were vaccinated with MOC2-CIITA (empty circles) did not develop cancer after 4 weeks, while all animals injected with MOC2-mock tumor cells (full squares) showed tumor development within 3 weeks. Mice were followed for tumor take (ordinate: percent of tumor-free mice) over time (abscissa) (**C**) MOC2-CIITA (empty circles) and MOC2-mock (full squares) growing tumors were measured for their tumor size (ordinate) over time (abscissa). p-values were obtained *via* unpaired Student t-test (**$p < 0.01$; ****$p < 0.0001$).

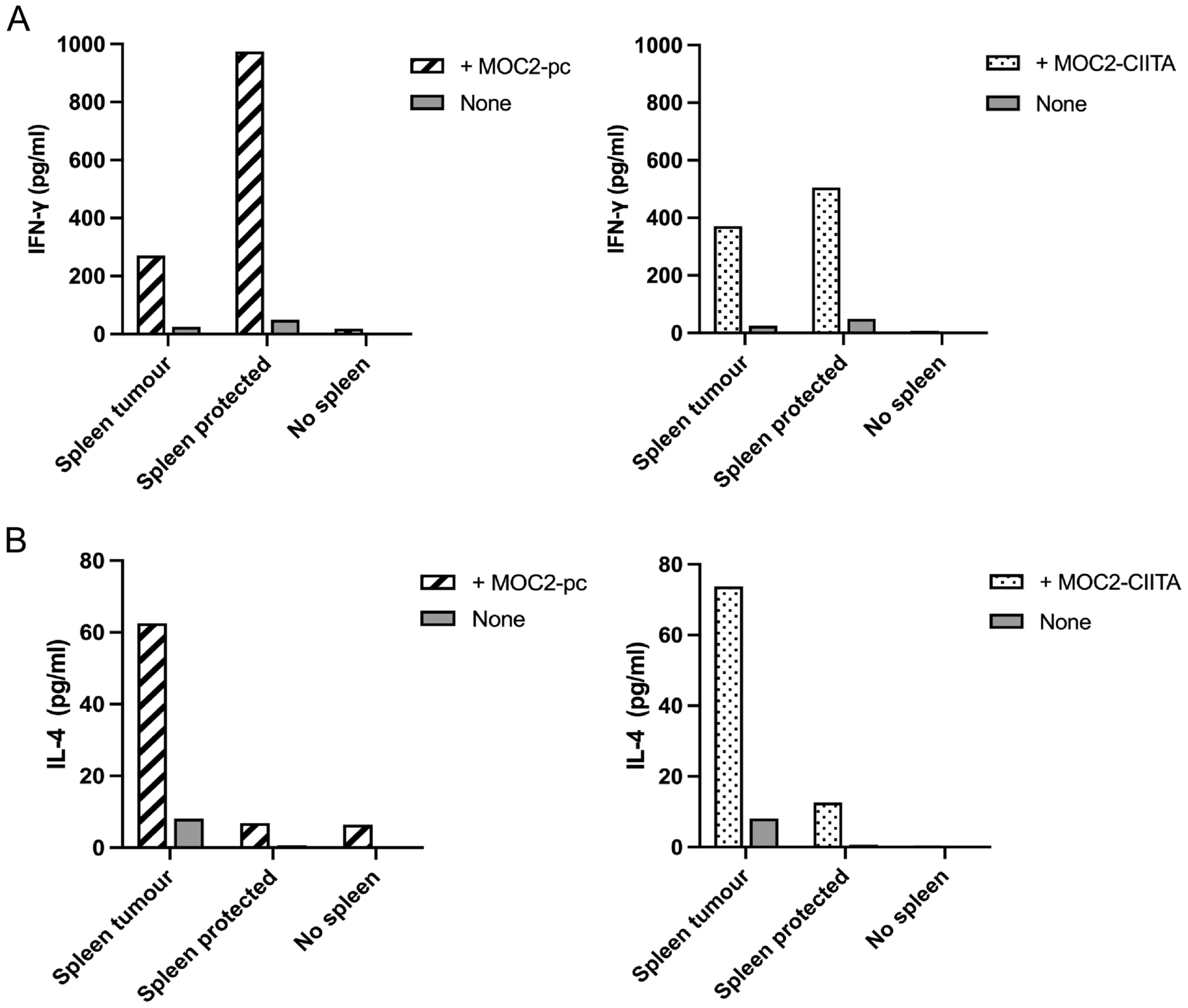

Figure S5. *In vitro assessment of TH phenotype.* The T cell immune response polarization was assessed at 4 weeks after tumor cells inoculation. Cytokines secretions was evaluated by *in vitro* stimulation of splenocytes isolated from MOC2-pc bearing mouse (Spleen tumor) or MOC2-CIITA vaccinated mouse (Spleen protected) with or without 50,000 cells of either MOC2-pc or MOC2-CIITA for 72 hours. As negative control, cytokines secretion of tumor cells alone was also evaluated (No spleen). IFN-γ **(A)** and IL-4 **(B)** secretions in the medium were measured by sandwich indirect ELISA. Representative results are shown in bar graphs.

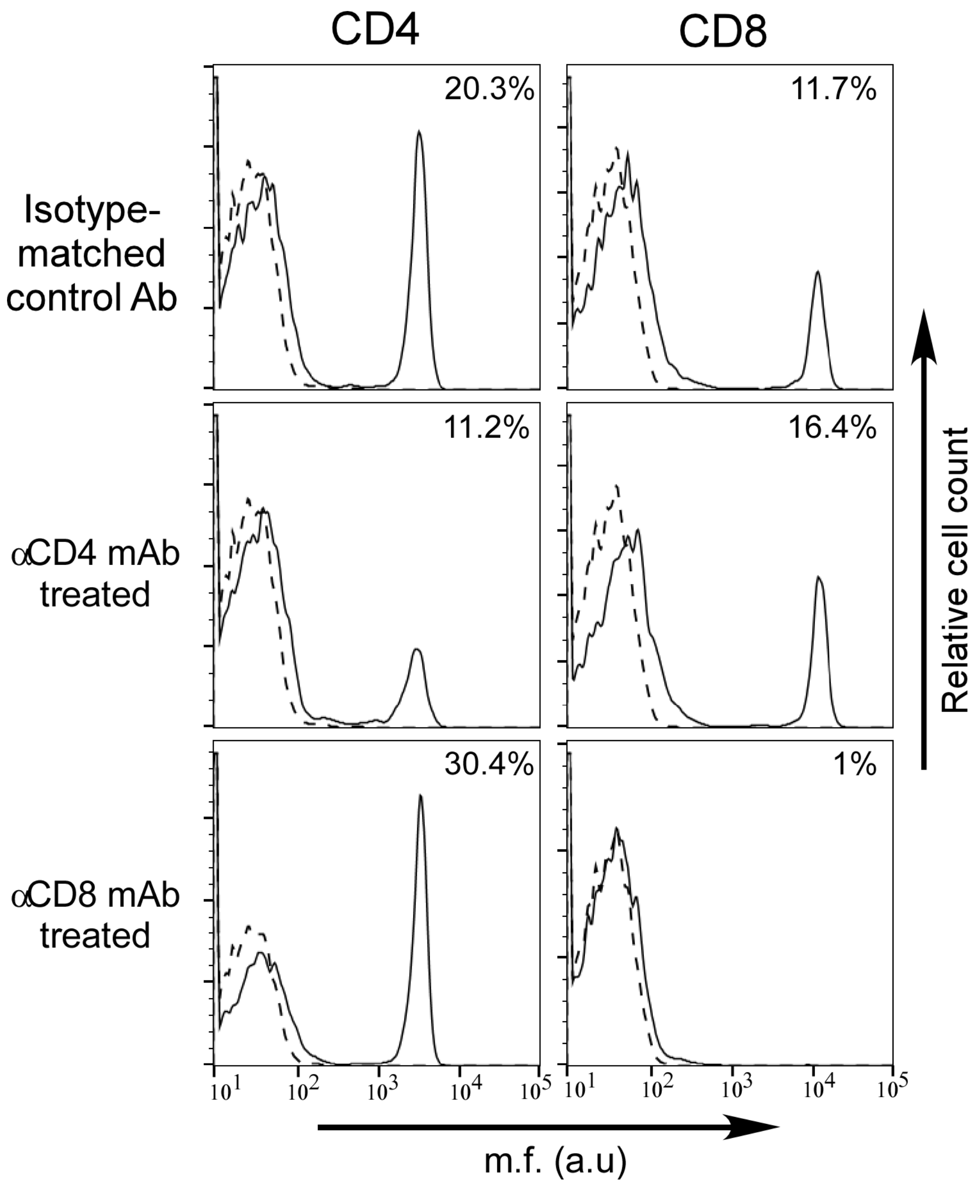

Figure S6. *Efficiency of both CD4+ and CD8+ T cells in vivo depletion*

The efficacy of $CD4^+$ and $CD8^+$ T cells depletion was assessed by immunofluorescence and flow cytometry on splenocytes derived from mice treated with anti-mouse CD4 (αCD4 mAb treated) and anti-mouse CD8 antibodies (αCD8 mAb treated) and compared with splenocytes isolated from mice treated with the isotype-matched control (isotype-matched control mAb). Anti RM4-5 anti-CD4 (BD, cat n° 550954), and 53-6.7 anti-CD8a (BioLegend, cat n° 100711) antibodies, were used to detect CD4+ and CD8+ T lymphocytes, respectively (solid lines). Controls (dashed line) were cells incubated with isotype-matched antibodies. Mean fluorescence (m.f.) values are expressed in the abscissa as arbitrary units (a.u.). The percentage of the specific T cells subpopulation is indicated in each panel (top right).
